# The Use and Effects of Electronic Health Tools for Patient Self-Monitoring and Reporting of Outcomes Following Medication Use: Systematic Review

**DOI:** 10.2196/jmir.9284

**Published:** 2018-12-18

**Authors:** Karla Lancaster, Aseel Abuzour, Manmeet Khaira, Annalise Mathers, April Chan, Vivian Bui, Annie Lok, Lehana Thabane, Lisa Dolovich

**Affiliations:** 1 Department of Family Medicine McMaster University Hamilton, ON Canada; 2 Leslie Dan Faculty of Pharmacy University of Toronto Toronto, ON Canada; 3 School of Pharmacy University of Waterloo Waterloo, ON Canada; 4 Department of Health Research Methods, Evidence, and Impact McMaster University Hamilton, ON Canada; 5 Pharmacy Department Sunnybrook Health Sciences Toronto, ON Canada; 6 Michael G. DeGroote National Pain Centre McMaster University Hamilton, ON Canada

**Keywords:** eHealth, mHealth, electronic health record, telemedicine, self-report, patient portals, patient-centered care, drug monitoring, adverse effects

## Abstract

**Background:**

Electronic health (eHealth) tools are becoming increasingly popular for helping patients’ self-manage chronic conditions. Little research, however, has examined the effect of patients using eHealth tools to self-report their medication management and use. Similarly, there is little evidence showing how eHealth tools might prompt patients and health care providers to make appropriate changes to medication use.

**Objective:**

The objective of this systematic review was to determine the impact of patients’ use of eHealth tools on self-reporting adverse effects and symptoms that promote changes to medication use. Related secondary outcomes were also evaluated.

**Methods:**

MEDLINE, EMBASE, and CINAHL were searched from January 1, 2000, to April 25, 2018. Reference lists of relevant systematic reviews and included articles from the literature search were also screened to identify relevant studies. Title, abstract, and full-text review as well as data extraction and risk of bias assessment were performed independently by 2 reviewers. Due to high heterogeneity, results were not meta-analyzed and instead presented as a narrative synthesis.

**Results:**

A total of 14 studies, including 13 randomized controlled trials (RCTs) and 1 open-label intervention, were included, from which 11 unique eHealth tools were identified. In addition, 14 RCTs found statistically significant increases in positive medication changes as a result of using eHealth tools, as did the single open-label study. Moreover, 8 RCTs found improvement in patient symptoms following eHealth tool use, especially in adolescent asthma patients. Furthermore, 3 RCTs showed that eHealth tools might improve patient self-efficacy and self-management of chronic disease. Little or no evidence was found to support the effectiveness of eHealth tools at improving medication recommendations and reconciliation by clinicians, medication-use behavior, health service utilization, adverse effects, quality of life, or patient satisfaction. eHealth tools with multifaceted functionalities and those allowing direct patient-provider communication may be more effective at improving patient self-management and self-efficacy.

**Conclusions:**

Evidence suggests that the use of eHealth tools may improve patient symptoms and lead to medication changes. Patients generally found eHealth tools useful in improving communication with health care providers. Moreover, health-related outcomes among frequent eHealth tool users improved in comparison with individuals who did not use eHealth tools frequently. Implementation issues such as poor patient engagement and poor clinician workflow integration were identified. More high-quality research is needed to explore how eHealth tools can be used to effectively manage use of medications to improve medication management and patient outcomes.

## Introduction

### Rationale

Use of the internet has increased considerably since the early 1990s. The World Bank reports that almost 44% of people across the globe used the internet in 2015, compared with 0.25% in 1993 [1]. This number is expected to increase to over 50% by 2019 [2]. Nearly two-thirds of internet users are estimated to access health information on the Web [3]. With such demand for accessible health information, electronic health (eHealth) has become a popular way to provide patients with health information, recommendations to self-manage their health, and access to their health records and data [3,4]. eHealth is defined as “an overarching term used today to describe the application of information and communications technologies in the health sector. It encompasses a whole range of purposes from purely administrative through to health care delivery” [5]. eHealth tools, therefore, are technologies that may include electronic medical records (EMRs), personal health records (PHRs), mobile apps, patient portals, information repositories, and many other internet-based programs or software used to help patients monitor and manage their health. eHealth tools may help decrease fragmentation of care by compiling patient health information from multiple providers into 1 easily accessible location [6], while also streamlining patient-provider communication and promoting shared decision making [3,4].

Well-functioning eHealth tools can help patients better understand their health [7] and may lead to improvements in patient-physician relationships [8]. eHealth tools can encourage patients to play a larger role in shared decision making and might increase focus on self-management and preventative care [8,9]. As technology advances, the use of eHealth tools can provide a level of convenience for both patients and providers [10]. These tools can generally be accessed from any internet-capable device and often provide a method of asynchronous communication such as emails and short message service (SMS) text messaging. These methods allow patients and providers to ask and answer questions at their convenience, creating less of a burden on physician workflow [8].

The ability of patients to use eHealth tools to better manage medication by reporting feedback on symptoms and use of medications directly to health care providers has not been comprehensively explored in the literature. Similarly, there is little evidence showing how eHealth tools might provide prompts to patients and health care providers to make appropriate changes to medication use based on this feedback. A synthesis of this literature will provide greater understanding of what eHealth tool design features may be helpful in patient reporting of medication-related experiences and outcomes.

### Objective

**Figure 1:**
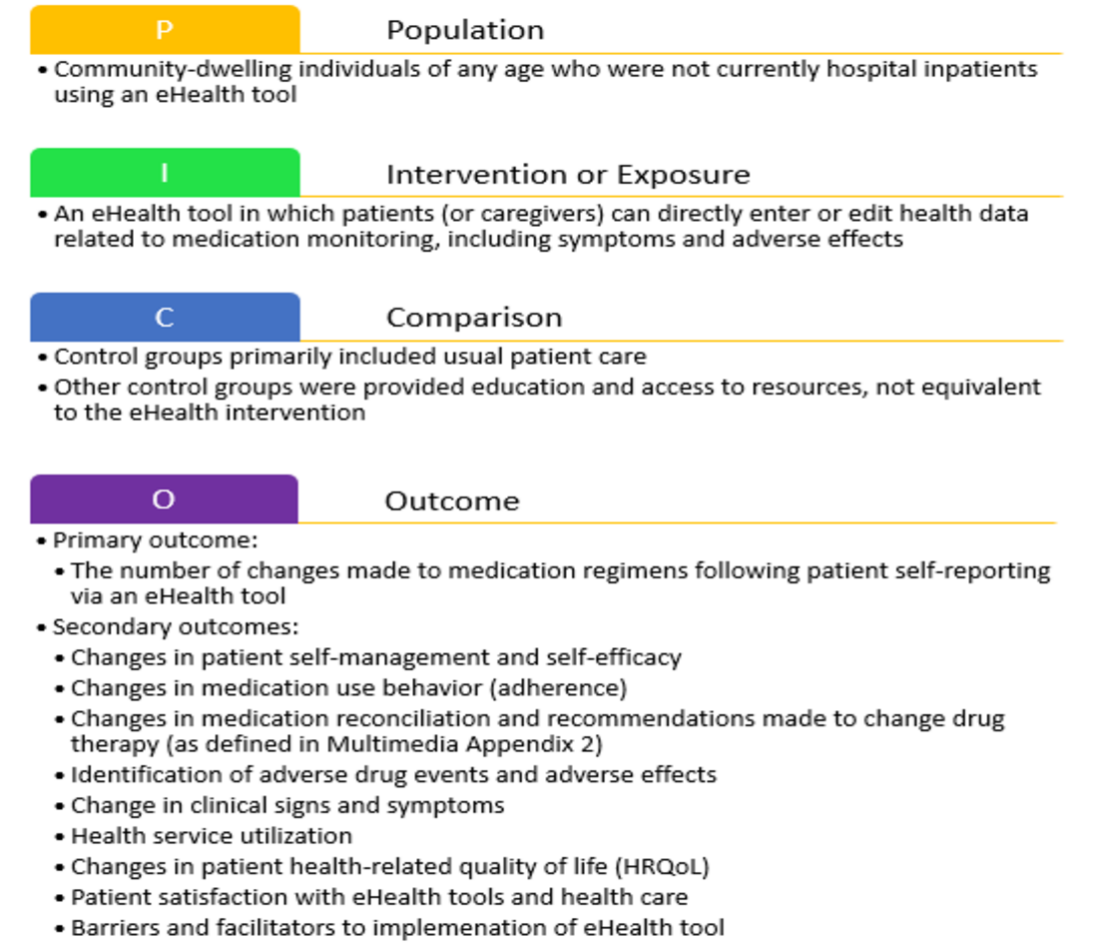
Use of the PICO model in this systematic review. eHealth: electronic health.

The objective of this systematic review was to determine the impact of patients’ use of eHealth tools on self-reporting adverse effects and symptoms that promote changes to medication use. The PICO model was used to focus the objective of the review, as seen in Figure 1.

## Methods

### Study Design and Study Selection

This systematic review was performed following steps outlined by Cochrane’s Effective Practice and Organization of Care group and reported based on the Preferred Reporting Items for Systematic Reviews and Meta-Analyses statement [11]. A total of 3 biomedical and health science databases were searched: MEDLINE/Ovid, EMBASE/Ovid, and CINAHL. References of all included articles were also searched. All 3 databases were searched from January 1, 2000, to April 25, 2018. The search was limited to articles published in English using terms representing eHealth (eg, Web-based applications), symptoms and adverse drug reaction reporting (eg, drug-related adverse effects and adverse reactions), and patient self-monitoring (eg, self-management; see Multimedia Appendix 1 for full search strategy and Multimedia Appendix 2 [12-17] for definitions for terminology used). The search date began from 2000, which generally marks the start of scientific reporting of eHealth interventions that would have relevance to the current use of eHealth tools. As a result of the aforementioned search strategy, studies were included in this review if they determined the effectiveness and impact of changes to medication regimens as a result of using eHealth tools. As such, this review investigated these effects using a comparative quantitative methodological approach.

### Criteria for Inclusion of Studies

For the purposes of this review, an eHealth tool was considered to be any internet-based intervention, including mobile health apps, used by patients for clinical purposes that focused on improving patient health and clinical outcomes. The term PHR refers to an eHealth tool wherein a patient has access to and can enter or edit their own health data. The population investigated was community-dwelling individuals of any age in an outpatient setting.

For a study to be included, the eHealth tool must have allowed patients (or caregivers) to enter information directly (as opposed to information being entered by a health care provider); included self-reporting functionalities focusing on medication monitoring, contain a medication monitoring or use component, or specifically incorporating the option for the patient or caregiver to enter symptoms including adverse effects; and needed to focus specifically on medication use, clinical outcomes, or symptom reporting following use of the eHealth tool. Any eHealth tools involving changes in medication reconciliation and recommendations made to changes in drug therapy were also included.

Exclusion criteria were conference abstracts; qualitative studies; articles without a comparator group; articles that did not report on at least one medication-related outcome; articles where self-management strategies focused on lifestyle modification, behavioral interventions, or nondrug interventions; articles focused solely on the validation of an eHealth tool; articles focused on methodological or technical aspects of eHealth interventions; articles containing nonempirical information; articles that synthesized information about multiple eHealth tools in an article (ie, review articles); and eHealth tools used by regulatory agencies to report adverse drug events (ADEs).

### Article Selection

**Figure 2:**
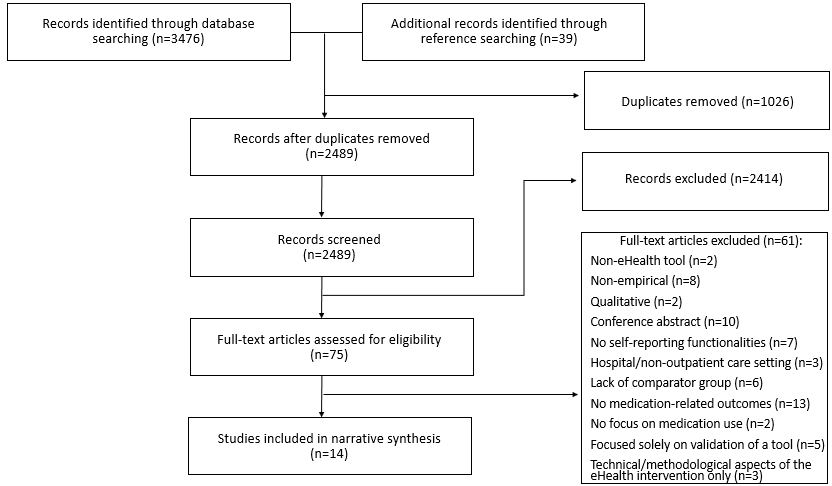
Preferred Reporting Items for Systematic Reviews and Meta-Analyses style study inclusion flowchart. eHealth: electronic health.

All potentially relevant articles were uploaded into DistillerSR software, which was used throughout the selection process. Potentially relevant articles underwent title, abstract, and full-text review. Articles that met inclusion criteria proceeded to data abstraction and risk of bias assessment. Articles not meeting inclusion criteria were excluded at both levels. Figure 2 represents the flow of articles through the selection process.

Title and abstract review were performed independently by 2 reviewers from a pool of 5 reviewers. Of these, 1 reviewer went through the reference lists of all the articles included in this study. Another reviewer went through reference lists of relevant systematic reviews identified during the literature search. Potentially relevant articles were identified. These articles went through abstract review by 2 reviewers. Studies found not to fit inclusion criteria after abstract review were excluded. Full-text review was performed independently by 5 reviewers. The kappa scores were calculated to determine agreement among reviewers who conducted review of titles and abstracts. All kappa scores calculated were greater than .93. Conflicts were resolved by consensus.

### Data Extraction and Risk of Bias Assessment

Data extraction and risk of bias assessment were performed for each study independently by 2 reviewers. Data extracted included study design and setting, participant demographics, number of participants in each group, intervention components, comparator group components, eHealth tool functionality measured, and results and significance levels for each outcome measure. Conflicts in data extraction were resolved by consensus. Risk of bias assessment used questions recommended by the Agency for Healthcare Research and Quality’s 2014 publication Methods Guide for Effectiveness and Comparative Effectiveness Reviews [18] and was performed for each study independently by 2 reviewers. All conflicts were resolved by consensus. The risk of bias assessment questions are presented in Multimedia Appendix 3.

### Outcomes and Analysis

The primary and secondary outcomes are listed in Figure 1. The primary outcome was the number of changes made to medication regimens following patient self-reporting via an eHealth tool. The included studies varied considerably in populations, eHealth tool functionality, outcomes measured, and study design. Due to high heterogeneity, meta-analysis of outcomes was not feasible. Therefore, results for each outcome were synthesized descriptively and presented as narrative. Available data on barriers to implementation were extracted from the article text and summarized qualitatively so as to heighten awareness of implementation issues.

### A Priori Subgroup and Sensitivity Analyses

Subgroup analyses were performed to investigate differences in treatment effect present because of (1) age of participants, (2) patients with specific conditions targeted by intervention, and (3) different features and functionalities of the included eHealth tools.

## Results

### Included Studies

A total of 3515 articles were generated from database and reference searching, resulting in 2489 potential articles that were screened based on their titles and abstracts, after duplicates were removed. Furthermore, 75 full-text articles were assessed for eligibility, of which 14 were included in this systematic review (see Figure 2 for more details).

Of the included articles, 13 were randomized controlled trials (RCTs) [19-31] and 1 was an open-label intervention [32]. A total of 10 studies were conducted in the United States [20-29], and 1 study was conducted in each of South Korea [19], Canada [30], Finland [31], and Denmark [32]. Dates of publication ranged from 2006 to 2017. The majority were published in 2007 or later (n=13). This distribution mirrors the increase in both internet and eHealth tool usage beginning in the late 2000s [1,33]. Further details on the characteristics of these studies can be seen in Table 1. Details regarding the design and outcomes of included studies are presented in Multimedia Appendix 4.

**Table 1:** Characteristics of included studies.

First author (year)	Country	Study design	Sample size	Patient age group study population
Cho (2006) [19]	South Korea	RCT^a^	80	Adults; adults (aged >30 years ) with type II diabetes
Chrischilles (2014) [20]	United States	RCT	1075	Elderly (aged >65 years); patients using a computer in the past month to visit websites or to send or receive email
Fiks (2015) [21]	United States	RCT	60	Children (aged 6 to 12 years) and parents
Grant (2008) [22]	United States	Cluster RCT	11 sites; 244 patients	Adults; adults with type II diabetes, A1c ≥7% or ≥1 diabetes medication, with ≥1 primary care visit in the last year and enrolled in Patient Gateway
Gustafson (2012) [23]	United States	RCT	305	Children (aged 4 to 12 years); patients with poorly controlled asthma and parents
Joseph (2007) [24]	United States	RCT	314	Children and young adults (ninth to eleventh grade); students with an asthma diagnosis or meeting asthma criteria
Joseph (2013) [25]	United States	RCT	422	Children and young adults (ninth to eleventh grade); students meeting asthma criteria or with an asthma diagnosis
Schnipper (2012) [26]	United States	Cluster RCT	11 sites; 541 patients	Adults; adults with ≥1 primary care visit and enrolled in Patient Gateway
Simon (2011) [27]	United States	RCT	208	Adults (aged >18 years); depressive disorder diagnosis with new antidepressant treatment
Weingart (2013) [28]	United States	RCT	738	Adults (aged 18 to 87 years); patients enrolled in PatientSite and received at least one new medication
Mooney (2017) [29]	United States	RCT	6 sites; 358 participants	Adults, seniors; English-speaking adults with a life expectancy of ≥3 months, beginning chemotherapy consisting of at least three cycles with daily access to a telephone
Ahmed (2016) [30]	Canada	RCT	2 sites; 100 participants	Adults (aged 18 to 69 years); French- or English-speaking adults diagnosed with asthma, prescribed at least one rescue medication, have poor asthma control, access to internet, and smoking <20 pack-years
Karhula (2015) [31]	Finland	RCT	517 participants (267 heart disease and 250 diabetes)	Adults, seniors; ability to complete questionnaires in Finnish, use the RPM system/devices, adequate cognition, able to walk; type 2 diabetes (diagnosed at least 3 months earlier) with hemoglobin A1c >6.5% within 1 year before screening; heart disease group (ischemic heart disease or heart failure)
Carlsen (2017) [32]	Denmark	Open-label	One site; 50 participants (29 electronic health tools, 21 control)	Children, adolescents; aged 10 to 17 years with ulcerative colitis or Crohn disease on maintenance infliximab treatment at the Department of Pediatrics, Hvidovre Hospital

^a^RCT: randomized controlled trial.

Of the 13 RCTs included in this review, 2 studies were cluster RCTs [22,26]. The remaining 11 RCTs used participants as the unit of randomization [19-21,23-25,27-31].

A total of 4 RCTs focused on pediatric and adolescent asthma patients [21,23-25]; 1 study focused on adult asthma patients [30]. Moreover, 1 trial included only elderly patients (aged 65 years and older) and focused on medication self-management and safety [20]. The remaining 7 trials all included both adult and elderly participants. From these studies, 1 focused on patients with depression [27]; 3 on patients with type 2 diabetes [19,22,31]; 2 on medication safety and use, including identification of ADEs [26,28] using eHealth tools; and 1 on identifying adverse effects in patients receiving chemotherapy [29].

In 3 studies, use of an eHealth tool in the intervention group was compared with usual care plus links to relevant websites [24,25,28]. Simon et al [27] compared their Web-based messaging eHealth tool for depression and Web-based messaging system to usual care with Web-based messaging between patients and health care providers. Gustafson et al [23] used nearly identical interventions in both groups; the control group was restricted from accessing the eHealth tool but participated in other aspects of the intervention (clinical visits, interviews, patient education, etc). Fiks et al [21] also used a usual care group with no access to the intervention Web portal; however, all health care providers had access to a computerized decision support system. Moreover, 2 studies [22,26] utilized a *double-dummy* style intervention, where both groups used Web-based PHRs to record information that differed only in content. Cho et al [19] compared an electronic blood glucose (BG) monitoring system with an informal paper-based monitoring system, with both groups receiving diabetes education and regular clinical visits. Chrischilles et al [20] utilized a conventional usual care group without supplementary information or resources. Mooney et al [29] used a self-monitoring tool to manage chemotherapy symptoms. Ahmed et al [30] developed an asthma portal to view patient’s personal health information, monitor patients, and provide feedback on self-management strategies. Karhula et al [31] used a management system for patient self-monitoring of diabetes. Only 1 study was identified as an open-label intervention study, which included a comparator group [32]. Carlsen et al [32] used an eHealth tool to monitor responses of patients with inflammatory bowel disease to determine the need to adjust treatment interval or dose.

### Quality of Included Studies

**Figure 3:**
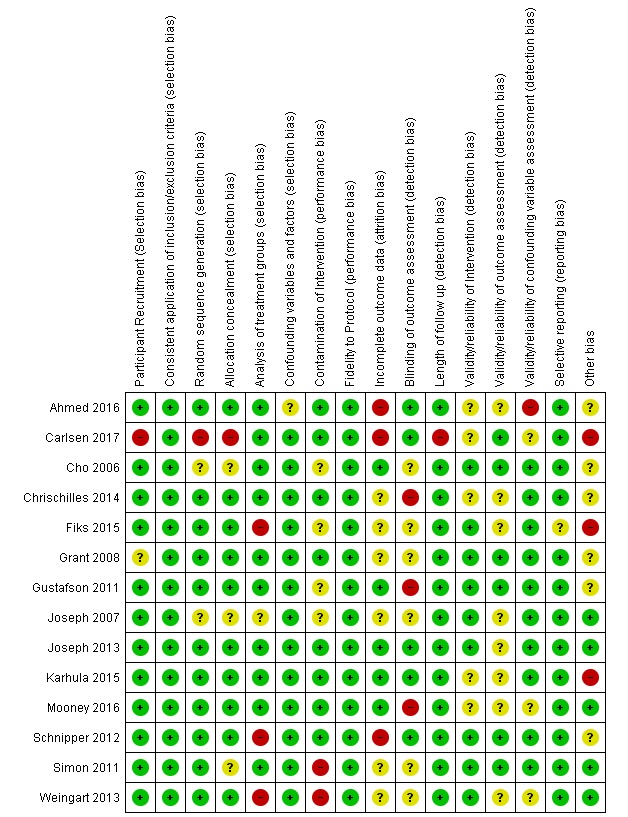
Summary chart of risk of bias assessment for included studies. Risk of bias summary: Green "+" symbols indicate a low risk of bias, yellow "?" symbols indicate an unknown risk of bias, and red "-" symbols indicate a high risk of bias.

Figure 3 displays a summary of the risk of bias assessment. The studies, overall, were of moderate quality, with studies ranging from poor to good. Common issues included small numbers of participants, lack of blinding, poor description of interventions, and contamination of intervention. Many trials relied only on patient self-reported data (as would be expected based on the topic), which can introduce bias if methods to ensure validity and reliability are not demonstrated.

### Types of Electronic Health Tools

From the 14 included studies, 11 unique eHealth tools were described. The 2 RCTs by Joseph et al [24,25] utilized the same asthma management eHealth tool. The cluster RCT by Schnipper et al [26] used a Web-based PHR to record information that differed only in content, which was nested within the larger RCT by Grant et al [22]. Two studies by Fiks and Ahmed used a Web-based portal for asthma symptom management [21,30]. Each study and eHealth tool is described in Multimedia Appendix 4. Features and functionalities of the eHealth tools are also presented in Table 2. Although Schnipper [26] and Grant [22] use the same eHealth tool, Schnipper’s study [26] investigates a specific medication module. Thus, they have been counted separately here.

All 11 eHealth tools from all 14 studies included a component where patients could self-report medication management information or changes, including symptoms, health data, adverse effects, or ADEs. A total of 12 studies included Web-based patient questionnaires or surveys [20,21,23-32]. Many studies used validated patient questionnaires but several developed their own. A list of patient questionnaires utilized by each study is presented in Table 3. In addition, 10 eHealth tools included patient educational resources [20,21,23-26,28-31]. Details of these resources are also listed in Table 3. Cho et al [19] measured outcomes by having a patient record their BG readings in the eHealth tool. Patients were also interviewed in person by clinicians every 3 months. Schnipper et al [26] had participants complete medication electronic journals, where they would note discrepancies between a Web-based medication list and their actual medications as taken. Karhula et al [31] used a 36-question survey for patients to report their health-related quality of life (HRQoL) score at baseline and post intervention.

### Outcomes of Included Studies

The results of each study by outcome can be seen in Multimedia Appendix 5.

#### Primary Outcome: Changes in Use of Medications and Other Therapies

A total of 6 RCTs [19-22,24,27] and 1 open-label intervention [32] measured this outcome. Moreover, 5 RCTs [20,22,24,27,32] found significant increases in medication changes as a result of using eHealth tools. All medication change outcomes reported were consistent with more appropriate prescribing and use of medications.

Chrischilles et al [20] found a significant reduction in use of more than 2 nonsteroidal anti-inflammatory drugs in the intervention group (14.1% vs 19%, *P*=.035). A nonsignificant trend approaching significance was seen for decreased number of over-the-counter medications used in the eHealth tool group (*P*=.05) [20]. Grant et al [22] found a significant increase in the number of diabetes mellitus–related medication changes in the intervention group (43.5 vs 6.2, *P*<.001). They also found that a significantly higher proportion of patients in the intervention group had medications initiated or dosages changed for hypertension (13% vs 0%, *P*=.02) and hyperlipidemia (11% vs 0%, *P*=.03).

**Table 2:** Features and functionalities of electronic health tools.

Topic; study author, year		Linked to electronic medical record		Function as personal health record		Clinicians can view self-reported information		Messaging between patient and clinician		Web-based surveys or questionnaires		Web-based drug list		Web-based access to lab results		Patient prompts or reminders		Patient educational resources	
**Focus on medication safety and usage**																			
	Chrischilles, 2014 [20]		✗^a^		✓^b^		✗		✗		✓		✓		✗		✓		✓
	Schnipper, 2012 [26]		✓		✓		✓		✓		✗		✓		✗		✗		✓
	Weingart, 2013 [28]		✓		✓		✓		✓		✓		?^c^		✓		✓		✓
**Focus on pediatric and adolescent asthma patients**																			
	Fiks, 2015 [21]		✓		✓		✓		✗		✓		✓		✗		✓		✓
	Gustafson, 2012 [23]		✗		✓		✓		✓		✓		✗		✗		✓		✓
	Joseph, 2007 and 2013 [24,25]		✗		✗		✗		✗		✓		✗		✗		✗		✓
**Focus on adult asthma patients**																			
	Ahmed, 2016 [30]		✓		✓		✓		✓		✓		✓		✗		✓		✓
**Focus on cancer patients**																			
	Mooney, 2017 [29]		✓		✓		✓		✗		✓		✓		✓		✓		✓
**Focus on diabetic patients**																			
	Cho, 2006 [19]		?		✓		✓		✓		✗		✓		✓		✗		✗
	Grant, 2008 [22]		✓		✓		✓		✓		✗		✓		✓		✗		✗
**Other**																			
	Simon, 2011 [27]		✓		✓		✗		✓		✓		?		✓		✗		✗
	Karhul, 2015 [31]		✓		✓		✓		✓		✗		✗		✗		✓		✓
	Carlsen, 2017 [32]		✗		✓		✓		?		✓		✗		✓		✓		✗

^a^✗ is used to demonstrate that the feature or functionality is not present and mentioned in the article.

^b^✓ is used to demonstrate that the feature or functionality is present and mentioned in the article.

^c^? is used to demonstrate that the feature or functionality is not discussed in the article.

**Table 3:** Use of patient questionnaires and educational resources in included studies.

First author (year)	Patient questionnaires used	Patient educational resources
Cho (2006) [19]	N/A^a^	N/A
Chrischilles (2014) [20]	Morisky adherence measure for medication adherence (modified); Assessing Care of Vulnerable Elders (ACOVE-3) medication-use quality indicators (modified); 12-item short form health survey (SF-12) for health status; other surveys developed by the study team [34,35]	ACOVE-3 adapted into patient medication safety messages [36]
Fiks (2015) [21]	Parent Patient Activation Measure; Integrated Therapeutics Group Child Asthma Short Form; Asthma Control Tool (ACT); other questions developed by the study team [37-39]	Handouts and videos available, but source and items used not reported
Grant (2008) [22]	Not reported by the study team, focused on medication adherence barriers	N/A
Gustafson (2012) [23]	Asthma Control Questionnaire; other questionnaires developed by the study team [40,41]	On the basis of the National Asthma Education and Prevention Program guidelines [42-44]
Joseph (2007) [24]	Lung Health Survey, developed by the study team, using items from the International Survey of Asthma and Allergies in Childhood questionnaire (ISAAC), and National Asthma Education and Prevention Program guidelines “Guidelines for the Diagnosis and Management of Asthma: Expert Panel Report II” (EPRII; adapted) [45,46]	EPRII; resources identified by Croft et al [46,47]
Joseph (2013) [25]	Multidimensional Scale of Perceived Social Support (adapted); Diagnosis Interview Schedule for Children Predictive Scales; Lung Health Survey, developed by the study team, using items from ISAAC; EPRII (adapted); EPRIII (adapted) [44-49]	EPRII; resources identified by Croft et al [46,47]
Schnipper (2012) [26]	Questionnaires developed by study team (adjudicated by physicians)	Source and items used not reported
Simon (2011) [27]	Hopkins Symptom Checklist; Patient Health Questionnaire (PHQ) Depression questionnaire; other questions developed by the study team [50-52]	N/A
Weingart (2013) [28]	Questions developed by the study team regarding new prescriptions and symptoms or adverse drug events	National Patient Safety Foundation website [53]
Mooney (2017) [29]	Questionnaire about symptoms severity for 11 symptoms related to chemotherapy	Self-management coaching provided based on symptoms; nurse practitioner follow-up, if required within 4 hours
Ahmed (2016) [30]	Mini-Asthma Quality of Life Questionnaire ; Chronic Disease Self-Efficacy Scale; ACT; Beliefs about Medicines Questionnaire; 9-item PHQ; EuroQol visual analog scale	MyAsthma Portal
Karhula (2015) [31]	SF-36 health survey	Patients provided a self-management guide. Additionally received health coaching phone calls
Carlsen (2017) [32]	IMPACT III (pediatric inflammatory bowel disease health-related quality of life measure); Total Inflammation Burden Score: (pediatric ulcerative colitis activity index/abbreviated Pediatric Crohn Disease Activity Index + fecal calprotectin)	N/A

^a^N/A: not applicable.

Joseph et al [24] found that a significantly higher proportion of those in the active group had a rescue inhaler available (39% vs 32%, *P*=.01). Simon et al [27] found that a significantly higher proportion of participants in the active group used antidepressants for an appropriate length of time (≥90 days; χ^2^_1_=10.5, *P*=.001). Carlsen et al [54] showed that eHealth tools might help identify instances where medication changes may be appropriate. Moreover, Carlsen et al [54] showed that the eHealth tool used resulted in at least one significant effect on changes to medication use; a significant difference was found between intervals of treatment for the eHealth group relative to the control group (2.35; 95% CI 1.5 to 3.2; *P*<.001) [54].

In contrast, Cho et al [19] found no significance in terms of total occasions of drug modification through the use of their eHealth tool intervention, internet-based glucose monitoring system (4.7 vs 5.5, *P*=.36). Fiks et al [21] provided descriptive evidence regarding the mean number of medications per child in both the intervention and control groups, yet no between-groups comparisons were made.

#### Secondary Outcome: Changes in Signs and Symptoms of Health Conditions

A total of 9 RCTs [19,21-25,27,30,31] and 1 open-label intervention [32] measured changes in signs and symptoms of health conditions. Of these, 8 reported at least one significant improvement in signs and symptoms [19,21,23-25,27,30,31]. Moreover, 4 studies found improvements in asthma symptoms [21,23-25], 1 found a significant reduction in hemoglobin A1c (HbA_1c_) [19], and 2 found a significant improvement in depression score [27,30].

Fiks et al [21] reported 17 instances of uncontrolled asthma in 13 children. They found that parents of active group children missed fewer days of work (mean of <0.1 vs 1.5, *P*=.001) and that the active group had less frequent flare-ups (mean of 1.4 vs 3.8, *P*=.02). Gustafson et al [23] found an increase in asthma control in the active group (mean change of −0.42 vs −0.11 on a 7-point Likert scale, *P*=.01).

Joseph et al [24] found that the active group had a lower risk for number of symptom nights (risk ratio [RR]=0.4, 95% CI 0.2 to 0.8, *P*=.009), symptom days (RR=0.5, 95% CI 0.4 to 0.8, *P*=.003), days of restricted activity (RR=0.5, 95% CI 0.3 to 0.8, *P*=.02), and school days missed (RR=0.3, 95% CI 0.1 to 0.7, *P*=.006). In another study, Joseph et al [25] reported a lower risk in the active group for symptom days (RR=0.8, 95% CI 0.6 to 1.0, *P*=.01). Following subgroup analysis, it was found that teenagers with moderate to severe asthma had fewer symptom days (RR=0.6, 95% CI 0.5 to 0.9, *P*=.01), total school days missed (RR=0.5, 95% CI 0.3 to 0.8, *P*=.009), school days missed because of asthma (RR=0.4, 95% CI 0.2 to 0.8, *P*=.007), and days of restricted activity (RR=0.6, 95% CI 0.4 to 0.9, *P*=.03).

Simon et al [27] reported a significant between-groups difference in mean depression score favoring the intervention group (mean score of 0.95 vs 1.17, *P*=.04). Ahmed et al [30] reported a statistically significant difference in depression using the Patient Health Questionnaire scale, as scores improved at 6 months (mean change −0.27, 95% CI −0.37 to −0.18 for a change of 5 units). Karhula et al [31] found no significant between-group difference in HbA_1c_ (change −0.106, 95% CI −0.33 to 0.11, *P*=.34); however, they did find a statistically significant decrease in waist circumference between intervention and control (change −1.711, 95% CI −3.042 to −0.38, *P*=.01). Cho et al [19] did find a decrease in mean HbA_1c_ in the active group (mean of 6.7% vs 7.4%, *P*=.006) at 30 months. Grant et al [22] found no differences between groups for HbA_1c_ and for percentage of patients at target HbA_1c_ levels. Similarly, Carlsen et al [32] found no significant difference for trough infliximab concentration when controlling for treatment intervals in the study (change of −2.19, 95% CI −5.37 to 0.99, *P*=.18).

#### Secondary Outcome: Patient Self-Management and Efficacy

A total of 5 RCTs measured this outcome [20,21,23,26,30]. In addition, 3 of the studies found increases in patient self-management or self-efficacy as a result of using eHealth tools [23,26,30].

Gustafson et al [23] found that self-efficacy had a positive, significant effect on Asthma Control Questionnaire (ACQ) scores (beta=.48, *P*=.01). They also found a positive significant effect of intervention on ACQ score when mediated by information competence (*τ*=−.235, *P*=.02). Schnipper et al [26] found that significantly more participants in the intervention group always disclosed drug therapy problems or new symptoms to clinicians (97.9% vs 87.1%, *P*<.001). Ahmed et al [30] found that a significant change in minimum asthma-related quality of life questionnaire adjusted for self-efficacy in adult asthma patients (0.24, 95% CI 0.16 to 0.32). Fiks et al [21] found that parents of children with asthma who used the eHealth tool improved their ability to manage asthma, although their findings were not statistically significant. In addition, they became more aware of the importance of ongoing attention to treatment. Chrischilles et al [20] found no difference between groups in ability to recognize adverse effects; however, in their as-treated analysis, they did find that high-frequency users had higher odds of recognizing symptoms and adverse effects (odds ratio, OR=1.76; 95% CI 1.08 to 2.86).

#### Secondary Outcome: Medication Use Behavior (Adherence)

A total of 3 RCTs measured this outcome, all using measures of medication adherence as a surrogate for medication use behavior [20,23,24]. None of these studies reported improvements over the 6 [20] and 12 months [23,24] studied.

#### Secondary Outcome: Medication Reconciliation and Recommendations to Change Drug Therapy

A total of 3 RCTs reported on this outcome [19,20,26]. Only Schnipper et al [26] found improvements in determining medication discrepancies when linking documented and patient-reported medication regimens using eHealth tools. Schnipper et al [26] explored the effects of a PHR medication module on medication accuracy and safety, reporting significantly lower odds of having discordant medications in the active group (OR=0.71, 95% CI 0.54 to 0.94, *P*=.01). In addition, Schnipper et al [26] found a significantly lower risk of discrepancies with the potential to cause severe harm in the active group (RR=0.31, 95% CI 0.10 to 0.92, *P*=.04). The number of medication discrepancies per patient with the potential for harm approached significance as a result of using eHealth tools (*P*=.05) [26].

Cho et al [19] acknowledged, using descriptive data, that a small percentage of individuals may have recommendations made for modification of drug therapy as a result of using their eHealth tool. Chrischilles et al [20] reported several instances of recommendations being made to alter drug therapy through medication reconciliation, none of which were significant.

#### Secondary Outcome: Adverse Effects and Adverse Drug Events

A total of 1 open-label intervention [32] and 5 RCTs measured this outcome [20,21,26,28,29]. Only Mooney et al [29] reported identification of adverse effects in favor of using eHealth tools, as there was a significant reduction in 10 of the 11 chemotherapy adverse effects in the intervention group (*P* value: .02 to <.001) relative to usual care. Descriptive evidence from Fiks et al [21] showed 1 instance of medication-related adverse effects. Carlsen et al [32] provided descriptive evidence that eHealth tools may lead to the identification of ADEs. The remaining 3 studies reporting on the identification of adverse effects [20] or ADEs [26,28] found no significant difference between intervention and control.

#### Secondary Outcome: Health Services Utilization

A total of 6 RCTs reported health service utilization outcomes [21,24,25,27,28,30]. Of these, only Joseph et al [24] found a significantly lower risk of hospitalizations as a result of using eHealth tools (RR=0.20, 95% CI 0.2 to 0.9, *P*=.01). The remaining 5 studies [21,25,27,28,30] found no difference between intervention and control groups in terms of health service utilization

#### Secondary Outcome: Patient Self-Reported Overall Health Status

A total of 4 studies measured this outcome [24,30-32], and none of the studies found differences in cumulative quality of life score between groups or a significant effect on overall health status.

#### Secondary Outcome: Patient Satisfaction With Health Care

A total of 2 RCTs [21,27] and 1 open-label intervention [32] measured this outcome. Only Simon et al [27] found that patient satisfaction improved as a result of using an eHealth tool. Simon et al [27] found that a significantly larger proportion of participants in the intervention group reported being very satisfied with the quality of their depression-related care (χ^2^_1_=8.38, *P*=.004). Fiks et al [21] found no significant changes when measuring this outcome, whereas Carlsen et al [32] provided positive descriptive evidence of patient and parent satisfaction using eHealth tools.

### Subgroup Analyses

A total of 4 RCTs investigated the use of eHealth tools in children and teens with asthma [21,23-25]. There is evidence that eHealth tools may have the potential to reduce symptoms of asthma, frequency of asthma flare-ups, and number of days of school or work missed because of asthma [21,23-25]. They may also promote better asthma control, availability and use of rescue inhalers, and may have the potential to improve asthma symptoms in vulnerable groups (ie, African-American adolescents living in urban centers) [24,25].

Subgroup analysis also found that multifaceted interventions combining use of eHealth tools with clinician support or case management and eHealth tools utilizing direct patient-provider communication might be more effective at improving some aspects of patient self-management and self-efficacy [23,26,30,31]. Both studies utilizing multifaceted interventions and direct patient-provider communication that measured these outcomes found positive significant results [23,26], whereas both studies using only eHealth tools with no patient-provider communication found no significant differences [20,21]. Detailed results from the subgroup analyses can be found in Multimedia Appendix 4.

#### Barriers to Implementation

Many studies reported barriers to the implementation of eHealth tools. The most common barrier was lack of participant engagement, resulting in low eHealth tool utilization rates. This was reported by 9 of the 14 studies [20,22-27,31,32]. A total of 3 studies noted distinct differences between high and low eHealth tool users [19,20,26], with high users generally seeing more improvements in health-related outcomes. Chrischilles et al’s study [20] was the only study to investigate the use of eHealth tools specifically in patients aged more than 65 years, and they found that patient engagement was negatively associated with age. Grant el al [22] found that patients with poor metabolic control were less likely to participate. The authors of several studies reported that a small sample size, high level of missing data, reduced power, and lower generalizability were observed as a result of low eHealth tool utilization and patient engagement [20,22,23,25-28,31,32]. Another important barrier, reported by 3 studies [22,26,29], was lack of clinician engagement and poor clinician training. This was generally because of time and workflow constraints [22,29] and lack of motivation [26,29]. Other implementation issues noted included lack of access to the internet [30], time burden of entering information [30], poor usability of eHealth tools [26], difficulties obtaining informed consent [24,25], and dilution of the intervention effect by the control group [23,26,28].

## Discussion

### Summary of Evidence

Evidence from 4 RCTs and 1 open-label intervention [20,22,24,27,32] show that eHealth tools focusing on symptom and adverse effect self-reporting can prompt positive changes in medication prescribing and use. In addition, the majority of eHealth tools studied were able to improve patient symptoms, regardless of functionalities, complexity, and differences in intervention. Those eHealth tools were particularly studied and found to be beneficial for improving signs and symptoms in children and adolescents with asthma [21,23-25]. This review supports the inclusion of patient entry or editing of symptoms into eHealth tools for the purposes of monitoring and reporting outcomes from the use of medications.

Evidence was found that eHealth tools improved the outcome of patient self-management and self-efficacy. Subgroup analysis found that eHealth tools that allow patients and clinicians to communicate directly, and multifaceted interventions combining eHealth tools with clinician support and case management might lead to greater increases in patient self-management and self-efficacy. It is notable that more significant improvements were found for more objective outcome measures, such as number of medication changes and clinical signs, and less were found for more subjective outcome measures such as self-management and self-efficacy. It may also be that sample sizes were too small to detect differences, particularly if this was not the primary objective for these studies. It is likely that the eHealth tools under investigation either did not provide effective content or functionalities to help participants improve self-management and medication management in participants or the tools used to measure these outcomes were not able to detect any differences between groups. Another possibility is the lack of patient understanding of chronic disease and poor perception of health goals.

It was thought that eHealth tools that focus on improvement of patient self-efficacy and self-management might lead to improved medication-use behavior, which in turn may lead to changes in medication use, identification of real or potential ADEs, improvement in signs and symptoms, and overall improvement in HRQoL. However, there is not enough evidence to draw conclusions as to the effectiveness of eHealth tools for identification of adverse effects, improving medication-use behavior, increasing recommendations to medication therapies and improving medication reconciliation, improving health service utilization, and improving overall health status and patient satisfaction. Only a small number of included studies investigated these outcomes; it is likely that with such a small overall sample size, it was not possible to find differences between groups.

### How Do These Results Compare With Other Reviews?

As with most systematic reviews on the subject of eHealth tools [7,8,33,54-57], this review found at best moderate evidence that patient reporting via eHealth tools can lead to improved clinical outcomes such as symptom reduction.

A 2012 systematic review by Ammenwerth et al found that use of patient portals linked to a PHR led to significant increases in medication adjustments in diabetic patients [55]. Other reviews and primary articles have also indicated that the use of eHealth tools may be more effective in specific patient populations such as patients with cancer [4,29,34,58]. This review found evidence that use of eHealth tools might increase the number of medication adjustments in diabetic patients [19,22]. In addition, patient-reported symptoms and adverse effects were used to identify toxicities in cancer patients, and in several instances, it lead to medication changes. It was also found that eHealth tools might improve signs and symptoms of asthma in children and teens [21,23-25].

Overall, this review supports findings by Ammenwerth et al [55] that interventions may be more effective at improving health outcomes if they combine eHealth tool features such as patient-provider communication and interactive coaching with eHealth tool use (see, eg, [21,22,26,28] as well as Table 2 and Multimedia Appendix 4 considering subgroup analyses). Evidence from this review indicates that eHealth tools in combination with clinician support or case management, and eHealth tools that encourage provider-patient communication may improve patient self-management and self-efficacy when compared with tools without these features [22,23,26].

### Strengths and Limitations

To our knowledge, this is the first systematic review of eHealth interventions focusing on patient self-reporting of symptoms and adverse effects. The review’s search strategy was augmented by reference searching. This review was limited to studies that included medication-related outcomes. The majority of studies included in this review were RCTs, most of which were of moderate quality.

This review also has a number of weaknesses. It was limited to studies published in English, which may have excluded relevant articles. No searching of grey literature was performed, as we focused on empirical work published in academic journals, and so it is possible that some early non–peer-reviewed reports may have been missed. We acknowledge the lack of definitional clarity surrounding the term *eHealth* and believe future research should focus on establishing better consensus for this term. There was considerable variety among the interventions in the studies, some of which included features such as direct health care provider follow-up, thus making it more complicated to determine which outcomes could be specifically attributable to using an eHealth tool. As this review also examined different populations of varying sample sizes and medical conditions for eHealth tools, it may be difficult to detect differences and generalize findings and conclusions. We did not include qualitative studies in our review because the goal of our study was to better understand the effectiveness and impact of changes to medication regimens based on quantifiable differences in using eHealth tools for self-reporting adverse effects and symptoms that promote changes to medication use versus a comparator. We value the insight of qualitative studies that have been investigated elsewhere [59,60]. Additional exploration of qualitative literature to better understand how use of these types of eHealth tools can generate impacts on medication use and health would be helpful.

### Implications for Practice and Future Research

Where possible, health care providers should encourage patient use of eHealth tools for symptom and adverse effect self-reporting. eHealth tools may be especially useful for reducing symptoms in certain populations, for example, children and teenagers with asthma. eHealth tools might also encourage patients to improve self-management behaviors and participate in shared decision making with clinicians. Having information from the EMR entered directly into the eHealth tool may reduce the burden on the patient to routinely update their clinical information (something that only highly motivated patients are likely to do regularly) [6]. Clinicians should be encouraged to communicate with patients via eHealth tools where possible, especially where patients are experiencing worsening of symptoms or medication-related adverse effects. Evidence suggests that using technologies such as mobile apps and SMS text messaging may improve patient engagement by allowing quick, convenient communication without a computer or internet connection [23,27]. Clinicians should be supported in their eHealth tool use, and interventions should focus on clinician training and engagement. Ensuring that interventions can be successfully incorporated into physician workflow is important [22,26].

There is a paucity of primary research articles investigating eHealth tools and their impact on medication use. Studies are generally small and of moderate quality. Large-scale RCTs focusing on the use of eHealth tools for medication and symptom management should be undertaken to establish more high-quality evidence. This is especially important given how ubiquitous the use of medication is. Furthermore, the effects of patient self-management and self-efficacy on medication use and symptom experience are not well studied; more research in this area could help drive creation of medication-focused eHealth tools. Low patient engagement and eHealth tool utilization were commonly noted implementation barriers; it could be that patients were not engaged in eHealth tool use enough for them to feel an impact on their satisfaction with health care or overall quality of life. Descriptive evidence shows low proportions of patients felt that eHealth tools improved their care or communication with providers, indicating that development of eHealth tools should focus on functionalities and outcomes that are important to the patient. This may be achieved by utilizing research on patient motivation and behavior change to increase patient engagement [20,24,25].

### Conclusions

The results of this review show initial and promising findings that specialized eHealth tools can be used for reporting and monitoring of symptoms and medication-related adverse effects and some evidence that use of eHealth tools have the potential to identify instances where changes in medication use may be appropriate. A modest amount of mixed evidence was found, demonstrating that eHealth tools can improve patient self-management and self-efficacy. Very little or no evidence was found to demonstrate that use of eHealth tools could increase numbers of medication recommendations or improve medication-taking behavior, health services utilization, identification of adverse effects, overall health status, and patient satisfaction. eHealth tools may be more effective at promoting medication changes and improving patient self-management and self-efficacy if they provide mechanisms for direct patient-provider communication and may be more effective in certain populations such as children and teenagers with asthma.

## Appendix

Multimedia Appendix 1 Search Strategy.

Multimedia Appendix 2 Definitions.

Multimedia Appendix 3 Risk of Bias Assessment Questions.

Multimedia Appendix 4 Supplemental Tables for Subgroups and Details about Outcomes.

Multimedia Appendix 5 Breakdown of Study Results by Outcome.

## Abbreviations

ACQ: Asthma Control Questionnaire
ADE: adverse drug event
BG: blood glucose
eHealth: electronic health
EMR: electronic medical record
HbA_1c_: hemoglobin A1c
HRQoL: health-related quality of life
OR: odds ratio
PHR: personal health record
RCT: randomized controlled trial
RR: risk ratio
SMS: short message service
