# Supplementary material for: The Use and Effects of Electronic Health Tools for Patient Self-Monitoring and Reporting of Outcomes Following Medication Use: Systematic Review
Source: J Med Internet Res. 2018 Dec 18;20(12):e294. doi: 10.2196/jmir.9284 (PMC6315271; doi:10.2196/jmir.9284)
Supplement: Multimedia Appendix 1 [file jmir_v20i12e294_app1.pdf]

Multimedia Appendix I:

Medline Search Strategy

April 25, 2018

1	electronic health records/
2	Health information exchange/
3	Health records, personal/
4	Web-based applications.mp.
5	ehealth.mp.
6	Ehealth.mp.
7	Online medical record*.mp.
8	Electronic medical record*.mp.
9	Electronic health record*.mp.
10	Interactive health communication applications.mp.
11	Personal health record*.mp.
12	Electronic personal health record.mp.
13	Self-management support system*.mp.
14	Telepharmacy.mp.
15	Electronic reporting system*.mp.
16	personal health record.tw.
17	ehealth.tw.
18	online medical record.tw.
19	electronic medical record.tw.
20	electronic health record.tw.
21	1 or 2 or 3 or 4 or 5 or 6 or 7 or 8 or 9 or 10 or 11 or 12 or 13 or 14 or 15 or 16 or 17 or 18 or 19 or 20

22	"Drug-Related Adverse effects and Adverse Reactions"/
23	Adverse drug reaction reporting/
24	Side effect*.mp.
25	Patient reported outcomes.mp.
26	Self-management.mp.
27	Patient monitoring.mp.
28	Patient self-assess*.mp.
29	22 or 23 or 24 or 25 or 26 or 27 or 28
30	21 and 29
31	smartphone.mp.
32	mobile phone.mp.
33	(self-care or self-report).kw.
34	(mobile health or mhealth).kw.
35	(telehealth or telemedicine).kw.
36	(mobile application or mobile apps).kw.
37	31 or 32 or 33 or 34 or 35 or 36
38	30 and 37
39	limit 38 to (english language and yr="2000-Current")

EMBASE search strategy

April 25, 2018

1	Electronic medical record/
2	Web-based applications.mp.

3	ehealth.mp.
4	Online medical record*.mp.
5	Electronic medical record*.mp.
6	Electronic health record*.mp.
7	Interactive health communication applications.mp.
8	Personal health record*.mp.
9	Electronic personal health record.mp.
10	Self-management support system*.mp.
11	1 or 2 or 3 or 4 or 5 or 6 or 7 or 8 or 9 or 10
12	Drug surveillance program/
13	Self-assessment.mp.
14	Drug related problem*.mp.
15	Self-monitor*.mp.
16	Patient reported outcomes.mp.
17	Self-management.mp.
18	patient self-assess*.mp.
19	12 or 13 or 14 or 15 or 16 or 17 or 18
20	11 and 19
21	smartphone.mp.
22	mobile phone.mp.
23	(self-care or self-report).kw.
24	(mobile health or mhealth).kw.

25	(telehealth or telemedicine).kw.
26	(mobile application or mobile apps).kw.
27	21 or 22 or 23 or 24 or 25 or 26
28	20 and 27
29	limit 28 to (english language and yr="2000-Current")

CINAHL search strategy

April 25, 2018

S38	S31 AND S38 Search modes - Boolean/Phrase<br>Limiters - Published Date: 20000101-20180425; English Language
S37	S31 OR S32 OR S33 OR S34 OR S35 OR S36
S36	smartphone
S35	mobile phone
S34	self-care OR self-report
S33	mobilehealth OR mealth
S32	telehealth OR telemedicine
S31	mobile applications OR mobile apps
S30	S16 AND S29
S29	S17 OR S18 OR S19 OR S20 OR S21 OR S22 OR S23 OR S24 OR S25 OR S26 OR S27 OR S28
S28	"patient self-assess*"

S27	"self-management"
S26	"patient reported outcomes"
S25	"self-monitor*"
S24	"drug effect*"
S23	"drug reaction"
S22	"drug related problem*"
S21	"side effect*"
S20	MH "Adverse Drug Event"
S19	MH "self disclosure"
S18	MH "self assessment"
S17	MH "self care"
S16	S1 OR S2 OR S3 OR S4 OR S5 OR S6 OR S7 OR S8 OR S9 OR S10 OR S11 OR S12 OR S13 OR S14 OR S15
S15	"Electronic reporting system*"
S14	"telepharmacy"
S13	"self-management support system*"
S12	"electronic personal health record"
S11	"personal health record*"
S10	"interactive health communication applications"
S9	"electronic health record*"

S8	"Electronic medical record*"
S7	"Online medical record*"
S6	"Ehealth"
S5	"ehealth"
S4	""Web-based applications""
S3	MH "Medical Records, Personal"
S2	MH "Electronic Data Interchange"
S1	MH "Computerized Patient Record"
