# Supplementary material for: The Use and Effects of Electronic Health Tools for Patient Self-Monitoring and Reporting of Outcomes Following Medication Use: Systematic Review
Source: J Med Internet Res. 2018 Dec 18;20(12):e294. doi: 10.2196/jmir.9284 (PMC6315271; doi:10.2196/jmir.9284)
Supplement: Multimedia Appendix 2 [file jmir_v20i12e294_app2.pdf]

Multimedia Appendix II: Definitions

EMRs

EMRs are defined by the Healthcare Information and Management Systems Society (HIMSS, 2006) as:

An application environment composed of the clinical data repository, clinical decision support, controlled medical vocabulary, order entry, computerized provider order entry, pharmacy, and clinical documentation applications. This environment supports the patient's electronic medical record across inpatient and outpatient environments, and is used by healthcare practitioners to document, monitor, and manage health care delivery within a care delivery organization (CDO). The data in the EMR is the legal record of what happened to the patient during their encounter at the CDO and is owned by the CDO [12].

EHRs

The definition of an EHR, as per HIMSS (2006), is:

A subset of each care delivery organization's EMR, presently assumed to be summaries [such as a] Continuity of Care Record (CCR) or…Continuity of Care Document (CCD), is owned by the patient and has patient input and access that spans episodes of care across multiple CDOs within a community, region, or state (or in some countries, the entire country)… The EHR can be established only if the electronic medical records of the various CDOs have evolved to a level that can create and support a robust exchange of information between stakeholders within a community or region [12].

Adverse effect

The definition of adverse effect, as per the WHO & Upsalla Monitoring Centre (2017), is:

A negative or harmful patient outcome that seems to be associated with treatment, including there being no effect at all [13].

Self-management

The definition of self-management is based on the following from Barlow (2001):

Self-management refers to the individual's ability to manage the symptoms, treatment, physical and psychosocial consequences and life style changes inherent in living with a [chronic condition]. Efficacious self-management encompasses ability to monitor one's condition and to effect the cognitive, behavioural and emotional responses necessary to maintain a satisfactory quality of life. Thus, a dynamic and continuous process of self-regulation is established [14].

Self-efficacy

Self-efficacy, as defined by Wood and Bandura (1998), is:

Beliefs in one's capabilities to mobilize the motivation, cognitive resources, and courses of action needed to meet given situational demands [15].

Medication Reconciliation

The definition of medication reconciliation is based on the Canadian Patient Safety Institute (2016):

Medication reconciliation is a formal process in which healthcare providers work together with patients, families, and care providers to ensure that accurate, comprehensive medication information is communicated consistently across transitions of care. It requires a systematic and comprehensive review of all the medications a patient is taking to ensure that medications being added, changed, or discontinued are carefully evaluated. A component of medication management, medication reconciliation informs and enables prescribers to make the most appropriate prescribing decisions for the patient [16].

Adverse drug event

The definition of adverse drug event, as per the Council of Europe's Expert Group on Safe Medication Practices:

Any injury occurring during the patient's drug therapy and resulting either from appropriate care, or from unsuitable or suboptimal care. Adverse drug events include: the adverse drug reactions during normal use of the medicine, and any harm secondary to a medical errors, both errors of omission or commission. [17].
