# Supplementary material for: The Use and Effects of Electronic Health Tools for Patient Self-Monitoring and Reporting of Outcomes Following Medication Use: Systematic Review
Source: J Med Internet Res. 2018 Dec 18;20(12):e294. doi: 10.2196/jmir.9284 (PMC6315271; doi:10.2196/jmir.9284)
Supplement: Multimedia Appendix 3 [file jmir_v20i12e294_app3.pdf]

Multimedia Appendix III: Risk of Bias Assessment Questions

Taken from the Agency for Health Research and Quality's 2014 Methods Guide for Effectiveness and Comparative Effectiveness Reviews [18]

Randomized Controlled Trials

Risk of Bias	Criterion
**Selection Bias**	Was the allocation sequence generated adequately (e.g., random number table, computer-generated randomization)?
	Was the allocation of treatment adequately concealed (e.g., pharmacy- controlled randomization or use of sequentially numbered sealed envelopes)?
	Were participants analyzed within the groups they were originally assigned to?
	Does the design or analysis control account for important confounding and modifying variables through matching, stratification, multivariable analysis, or other approaches?
**Performance Bias**	Did researchers rule out any impact from a concurrent intervention or an unintended exposure that might bias results?
	Did the study maintain fidelity to the intervention protocol?
**Attrition Bias**	If attrition (overall or differential nonresponse, dropout, loss to follow-up, or exclusion of participants) was a concern, were missing data handled appropriately (e.g., intention-to-treat analysis and imputation)?
**Detection Bias**	In prospective studies, was the length of follow-up different between the groups, or in case-control studies, was the time period between the intervention/exposure and outcome the same for cases and controls?
	Were the outcome assessors blinded to the intervention or exposure status of participants
	Were interventions/exposures assessed/defined using valid and reliable measures, implemented consistently across all study participants?
	Were outcomes assessed/defined using valid and reliable measures, implemented consistently across all study participants?
**Reporting Bias**	Were the potential outcomes pre-specified by the researchers? Are all pre-specified outcomes reported?

Cohort and Non-Randomized Controlled Trials

Risk of Bias	Criterion
**Selection Bias**	Were participants analyzed within the groups they were originally assigned to?
	Did the study apply inclusion/exclusion criteria uniformly to all comparison groups?
	Did the strategy for recruiting participants into the study differ across study groups?
	Does the design or analysis control account for important confounding and modifying variables through matching, stratification, multivariable analysis, or other approaches?
**Performance Bias**	Did researchers rule out any impact from a concurrent intervention or an unintended exposure that might bias results?

	Did the study maintain fidelity to the intervention protocol?
**Attrition Bias**	If attrition (overall or differential nonresponse, dropout, loss to follow-up, or exclusion of participants) was a concern, were missing data handled appropriately (e.g., intention-to-treat analysis and imputation)?
**Detection Bias**	In prospective studies, was the length of follow-up different between the groups, or in case-control studies, was the time period between the intervention/exposure and outcome the same for cases and controls?
	Were the outcome assessors blinded to the intervention or exposure status of participants
	Were interventions/exposures assessed/defined using valid and reliable measures, implemented consistently across all study participants?<br>Were outcomes assessed/defined using valid and reliable measures, implemented consistently across all study participants?
	Were confounding variables assessed using valid and reliable measures, implemented consistently across all study participants?
**Reporting Bias**	Were the potential outcomes pre-specified by the researchers? Are all pre-specified outcomes reported?
