# Supplementary material for: The Use and Effects of Electronic Health Tools for Patient Self-Monitoring and Reporting of Outcomes Following Medication Use: Systematic Review
Source: J Med Internet Res. 2018 Dec 18;20(12):e294. doi: 10.2196/jmir.9284 (PMC6315271; doi:10.2196/jmir.9284)
Supplement: Multimedia Appendix 4 [file jmir_v20i12e294_app4.pdf]

Multimedia Appendix IV: Supplemental Tables for Subgroups and Details about Outcomes.

Supplemental Table 1: Design and outcomes of included studies

1st Author (year)	Intervention	Comparator Group(s)	Study Duration	Outcomes Measured	Notable ehealth Tool Features:
**Cho (2006) [19]**	IBGMS<br><br>Participants attended an initial diabetes educational session<br><br>Outpatient clinical visit for diabetes every three months<br><br>Use of IBGMs website to upload blood glucose, medication use, blood pressure and weight data	Control group participated in the initial diabetes educational session and attended clinical visits every three months<br><br>Used informal paper-based system to record clinical outcomes	30 months	• Recommendations provided for drug modification<br>• Total number of drug modifications	Participants able to ask health questions via the website
**Chrischilles (2014) [20]**	Iowa PHR<br><br>Designed for older adults, focusing on simplicity, readability, and quick navigation<br><br>Patients manually enter information (such as medications)	Usual care	6 months	• Mean number of prescription medications<br>• Mean number of OTC medications<br>• Change in medication use in past three months<br>• Use of potentially inappropriate medications (ACOVE)<br>• Taking two or more NSAIDS or ASA<br>• Number of medication management problems<br>• Knows how to recognize adverse effects<br>• Adverse effects in past three months<br>• Modified Morisky adherence score<br>• Keep list of current medications<br>• Reason for medications on list<br>• Usually shows drug list to doctor<br>• Put OTC drugs on list<br>• Updated list in past three months	Health event tracking, drug alerts

**Fiks (2015) [21]**	MyAsthma<br><br>Tracks families' asthma treatment concerns and goals, children's asthma symptoms, medication adverse effects, and adherence	Access to a clinician-focused decision support system	6 months	• Medication receipt<br>• Asthma-related healthcare use<br>• Number of adverse effects reported<br>• Number of poor/uncontrolled asthma<br>• Nighttime symptoms<br>• Daytime symptoms<br>• Functional limitations<br>• Parent activation<br>• Satisfaction with asthma care<br>• Days of school missed in past month (child)<br>• Days of work missed in past month (parent)	Symptom and adverse effect tracking, goal planning, clinical decision support
**Grant (2008) [22]**	Patient Gateway<br><br>Online PHR with access to lab results and medication lists<br><br>Patients reviewed drug lists, reported discrepancies, ask/answer questions<br><br>Generate diabetes care plan	Control patients were asked to review medical histories, cancer screening history, and other non-diabetes related tasks similar to the intervention group	12 months	• Diabetes related medication changes<br>• Medications initiated or changed for specific conditions<br>• HbA1C<br>• % of patients achieving goal HbA1C	Ability to refill medications online
**Gustafson (2012) [23]**	Comprehensive Health Enhancement Support System plus monthly nurse case management<br><br>(CHESS+CM) System for parents and children	Usual care plus asthma information	12 months	• Medication adherence<br>• Competence<br>• Self-efficacy<br>• Social support<br>• HRQoL<br>• Symptom free days	Information presented as games
**Joseph (2007) [24]**	Puff City<br><br>Web-based program focused on controller medication adherence, rescue inhaler availability, and smoking cessation/reductio	Four thirty-minute sessions of generic asthma education websites	12 months	• Asthma-related ED visits<br>• Asthma-related hospitalizations<br>• Controller medication adherence<br>• QoL<br>• Symptom-nights<br>• Symptom-days<br>• Days of restricted activity	Module to visually identify drugs and dosage

	n			• Days of changed plans<br>• Schooldays missed<br>• Changes in rescue inhaler availability	
**Joseph (2013) [25]**	Puff City<br><br>Web-based program focused on controller medication adherence, rescue inhaler availability, and smoking cessation/reduction	Four thirty-minute sessions of generic asthma education websites	12 months	• ED visits and hospitalizations<br>• Symptom-days<br>• Symptom-nights<br>• Schooldays missed<br>• Days of restricted activity	Module to visually identify drugs and dosage
**Schnipper (2012) [26]**	Patient Gateway<br><br>Online PHR with access to medication lists<br><br>Intervention group filled out medication ejournals<br><br>Contacted by study team to report medication discrepancies, ADE and symptoms	Active Controls filled out ejournals relating to medical history and health maintenance. Matched controls also contacted by study team to report medication discrepancies, symptoms, and ADEs	18 months	• % discordant medications<br>• Discrepancies with medication list<br>• Discrepancies with potential for harm<br>• Discrepancies with potential for severe harm<br>• Preventable/ameliorable ADEs<br>• Duration of ameliorable ADEs<br>• Reporting of all prescribed medications<br>• Reporting of medication-related problems or new symptoms	Online prescription refills, appointments, referrals
**Simon (2011) [27]**	EpicCare<br><br>Patient website linked to this medical record able to refill prescriptions, view laboratory results and exchanging online messages with providers	Usual care	5 months	• Number receiving additional antidepressant<br>• Number (%) making any specialty mental health visits<br>• Number using antidepressant for over 90 days<br>• Mean mental health visits for medication management<br>• Quality of antidepressant treatment<br>• Severity of depression<br>• Outcome and mean SCL at follow-up	Ability to refill medications online
**Weingart**	PatientSite	Message with	3 months	• Number of ADEs	Online

**(2013) [28]**	Web-based patient portal accessible to patients, allowing electronic messaging between patients and providers	a link to the National Patient Safety Foundation Web site with information about resources for patients and families		• Preventable ADEs<br>• Ameliorable ADEs<br>• Healthcare use	prescription refills, appointments, referrals
**Mooney (2017) [29]**	Symptom Care at Home (SCH) Interactive voice response system with four components: monitoring of 11 symptoms through self-reporting, self-management coaching, automated alerts for poorly controlled symptoms, and an electronic symptoms-based guideline for NPs to follow-up.	Enhanced Usual Care (UC) Includes the symptom reporting component of SCH but lacks self-management coaching and NP intervention	From study entry to through chemotherapy course completion or 6 months, whichever came first	• Symptom severity across symptoms and the number of severe across symptoms and the number of severe, moderate, mild, and no symptom days	Graphing of symptom history in EHR
**Ahmed (2018) [30]**	My Asthma Portal (MAP) View their personal health information, access educational information, monitor and receive feedback regarding self-management strategies	Usual Care No access to MAP – continued to receive ongoing asthma care from their pulmonologis t	6 months	• Asthma control at six months<br>• Asthma Quality of life	Ability to communicate with nurse case manager through the MAP
**Karhula (2015) [31]**	Remote Patient Monitoring (RPM) System: PHR App Enter steps, weight, blood pressure and blood glucose, sent to clinician, has reminders and messaging	Standard Care Disease management information booklet at time of diagnosis. Standard lab coaching and appointments	12 months	• Primary outcomes are HRQL and HbA1c<br>• Secondary outcomes include BP, weight, waist circumference, triglycerides, total cholesterol, LDL, HDL and adherence.	Patient sends info to PHR weekly which links to EHR
**Carlsen (2017) [32]**	eHealth tool Completed TIBS to determine	Control group No use of the eHealth tool	Varied, max follow up is 2 years	• Primary outcomes surround treatment: interval of drug therapy	eHealth tools calculates total inflammation

	frequency of infusions. Patients entered symptom scores			and treatment dose<br>• Secondary outcomes measure IFX antibodies and concentration	burden score weekly to determine timing of next visit based on symptoms entered and fecal calprotectin analysis

Supplemental Table 2: Results per outcome from included studies-- Primary Outcome: Changes in Use of Medications and Other Therapies

Study	Outcome measure	Effect measures	Result
Cho, 2006 [19]	Total occasions of drug modification (Reports through IBGMS)	n<br>Mean times/person (SD)	Intervention:<br>150<br>4.7 (2.9)<br><br>Control:<br>187<br>5.5 (4.7)<br><br>t = 0.92, *P* = .36
Chrischilles, 2014 [20]	Number of prescription medications (Patient self-report of medications used)	Mean (SD)	Intervention: 4.0 (3.1)<br>Control: 4.1 (3.2)<br>*P* = .68
	Number of OTC medications (Patient self-report of medications used)	Mean (SD)	Intervention: 3.6 (2.5)<br>Control: 3.9 (2.7)<br>*P* = .053
	Any change in medication use in past 3 months (Patient self-report of medications used)	n (%)	Intervention: 349 (43.5)<br>Control: 124 (45.4)<br>*P* = .58
	Use of potentially inappropriate medications (Patient self-report of medications used)	n (%)	Intervention: 164 (20.4)<br>Control: 53 (19)<br>*P* = .71
	Taking 2 or more NSAIDS (including aspirin) (Patient self-report of medications used)	n (%)	Intervention: 113 (14.1)<br>Control: 53 (19)<br>*P* = .036
Fiks, 2015 [21]	Medication receipt (EHR listed asthma medications)	Mean number medications / child	Intervention:<br>Inhaled steroids: 1.1<br>Oral steroids: 0.4<br><br>Control:<br>Inhaled steroids: 0.7<br>Oral steroids: 1.0<br><br>No between-groups comparisons reported
Grant, 2008 [22]	Diabetes-related medication changes (Patient self-report via PHR)	n (%)	Intervention: 43.5 (53)<br>Control: 6.2 (15)<br>*P* < .001
	Hypertension-related medication changes	n (%)	Intervention: 11 (13)<br>Control: 0 (0)<br>*P* = .02
	Hyperlipidemia-related medication changes	n (%)	Intervention: 9 (11)<br>Control: 0 (0)<br>*P* = .03
Joseph, 2007 [24]	Rescue inhaler availability (patient self-report)	n (%)	Intervention: 59 (39)<br>Control: 46 (32)<br>*P* = .01
Simon, 2011 [27]	Participants receiving additional antidepressant	n (%)	Intervention: 23 (22)<br>Control: 16 (16)

			P = .27
	Participants using antidepressant for over 90 days	n (%)	Intervention: 86 (81)<br>Control: 62 (61)<br>*P* = .001
Carlsen, 2017 [32]	Difference in average IFX dose per treatment between ehealth group and control	Dose in mg/kg (95% CI)	0.54 (-0.36 to 1.43)<br>*P = 0.24*
	Difference in treatment interval between ehealth group and control	Weeks (95% CI)	2.35 (1.5 to 3.2)<br>*P < 0.001*

Supplemental Table 3: Results per outcome from included studies-- Secondary Outcome: Patient Self-Management and Efficacy

Study	Outcome measure	Effect measures	Result
Chrischilles, 2014 [20]	Ability to recognize adverse effects (patient self-report)	n (%)	Intervention: 566 (73.7)<br>Control: 201 (75.3)<br>*P* = .61
	Usually shows medication list to doctor (patient self-report)	n (%)	Intervention: 435 (78.2)<br>Control: 154 (78.6)<br>*P* = .92
Fiks, 2015 [21]	Parent Activation Measure questionnaire	Mean (SD)<br><br>Between- groups change score	Intervention: 78.1 (72.6)<br>Control: 83.1 (15.9)<br>0.1<br>*P* = .922
Gustafson, 2012 [23]	Self-efficacy (patient self-report)	alpha (*P*) (intervention effect on the mediator)<br>beta (*P*)(mediator's effect on dependent variable).<br>tau (*P*) (Intervention effect on the dependant variable after mediational test)	alpha = .080, *P* = .14<br><br>beta = .476, *P* = .01<br><br>tau = -.182, *P* = .07
	Information competence (patient self-report)	alpha (*P*)<br>beta (*P*)<br>tau (p)	alpha = .079, *P* = .09<br>beta = .476, *P* = .64<br>tau = -.235, *P* = .02
Schnipper, 2012 [26]	Report medication-related problems or new symptoms to doctor (patient self-report)	n (%) who responded "yes"	Intervention: 236 (97.9)<br>Control: 149 (87.1)<br>*P* < .001
Ahmed, [30]	Results for differences in minimum asthma quality of life questionnaire adjusted for self-efficacy	Mean change (95% CI)	0.24 (0.16 to 0.32)

Supplemental Table 4: Results per outcome from included studies-- Secondary Outcome: Medication Use Behaviour (Adherence)

Study	Outcome measure	Effect measures	Result
Chrischilles, 2014 [20]	Modified Morisky adherence score (patient self-report)	Mean (SD)	Intervention: 13.8 (1.9)<br>Control: 13.9 (1.9)<br>*P* = .9821
Gustafson, 2012 [23]	Composite adherence score (sum of self-reported adherence data and pharmacy refill data)	Mean change in intervention group minus mean change in control group (95% CI)	1.48% (-8.15% to 11.11%)<br>*P* = .76
	Pharmacy refill possession ratio	Mean change in intervention group minus mean change in control group (95% CI)	-3.95% (-11.26% to 16.87%)<br>*P* = .69
	Self-report of inhaled corticosteroid use	Mean change in intervention group minus mean change in control group (95% CI)	11.64% (-8.65% to 13.93%)<br>*P* = .65
	Self-report of anticholinergic use	Mean change in intervention group minus mean change in control group (95% CI)	2.81% (-11.26% to 16.87%)<br>*P* = .69
Joseph, 2007 [24]	Controller medication adherence (patient self-report)	n (%)	Intervention: 20.4 (31)<br>Control: 12.6 (18)

			P = .09
Karhula, 2015 [31]	Average number of calls per patient<br>Number of self-reported measurements<br>Weight reports<br>Step counts<br>BP reports<br>Blood glucose reports<br>Health-related quality of life assessments<br>Heart disease group<br>Diabetes group	N (SD)	8.7 (1.6)<br>8.5 (1.9)<br>P=0.40
	Time consumed by the nurse after the coaching in minutes<br>Heart disease group<br>Diabetes group	N (SD)	3.8 (3.0)<br>4.5(3.6)<br>P<0.001
	Mean duration of coaching in minutes<br>Heart disease group<br>Diabetes group	Mean duration of coaching in minutes	N(SD)<br>19.2 (8.1)<br>P=0.004
	Mean time consumed by nurse for preparation of the call in minutes<br>Heart disease group<br>Diabetes group	N (SD)	3.5(2.5)<br>4.2(3.2)<br>P<0.001

Supplemental Table 5: Results per outcome from included studies-- Secondary Outcome: Medication Reconciliation and Recommendations to Change Drug Therapy

Study	Outcome measure	Effect measures	Result
Cho, 2006 [19]	Recommendations provided for drug modification (reports through IBGMS)	n (%)	192 (12.1) (descriptive data only)
Chrischilles, 2014 [20]	Number of medication management problems (patient self-report of medication management problems)	Mean (SD)	Intervention: 1.4 (1.4)<br>Control: 1.6 (1.5)<br>*P* = .1514
	Keep list of current medications (patient self-report of medication management behaviour)	ITT:<br>n (%)<br><br>As-treated Analysis (ATA):<br>n (%)<br>OR (95% CI)	ITT:<br>Intervention: 559 (70.6)<br>Control: 196 (72.1)<br>*P* = .6432<br><br>ATA:<br>High freq users: 113 (88.3)<br>Low freq users: 446 (67.2)<br>3.68 (1.83 to 7.37)
	Reason for medications on list (patient self-report of medication management behaviour)	ITT:<br>n (%)<br><br>ATA:<br>n (%)<br>OR (95% CI)	ITT:<br>Intervention: 210 (38.7)<br>Control: 59 (30)<br>*P* = .064<br><br>ATA:<br>High freq users: 57 (50)<br>Low freq users: 513 (34.6)<br>2.14 (1.13 to 3.64)
	Put OTC drugs on list (patient self-report of medication management behaviour)	n (%)	Intervention: 435 (78.1)<br>Control: 155 (79.1)<br>*P* = .77
	Updated list in past three months (patient self-report of medication management behaviour)	n (%)	Intervention: 293 (52.9)<br>Control: 105 (54.4)<br>*P* = .72
	Discrepancies between doctor and patient medication lists	ATA:<br>n (%)<br>OR (95% CI)	High freq users: 24 (19)<br>Low freq users: 53 (8)<br>2.21 (1.27 to 3.85)
Schnipper, 2012 [26]	Discordant medications (Patient self-report survey assessed by RA or blinded physician adjudicators)	% mean (SD)<br><br>Adjusted OR (95% CI),	Intervention: 42% (29%)<br>Control: 51% (30%)<br>*P* < 0.001<br><br>0.71 (0.54 to 0.94)<br>*P* = .01
	Number of discrepancies per patient with potential for harm (Patient self-report survey assessed by RA or blinded physician adjudicators)	Mean (SD)<br><br>Adjusted RR (95% CI),	Intervention: 0.24 (0.62)<br>Control: 0.34 (0.85)<br>*P* = .23<br><br>0.65 (0.42 to 1.02)<br>*P* = .059
	Number of discrepancies per patient with potential for severe harm (Patient self-	Mean (SD)	Intervention: 0.03 (0.22)<br>Control: 0.08 (0.35)<br>*P* = 0.03

	report survey assessed by RA or blinded physician adjudicators)	Adjusted RR (95% CI),	0.31 (0.10 to 0.92)<br>*P* = .04
	Report all prescribed medications (Patient self-report at follow-up survey)	Number who responded "always"/Number who answered that question (%)	Intervention: 515/689 (74.8)<br>Control: 399/572 (69.8)<br>*P* = .048

Supplemental Table 6: Results per outcome from included studies-- Secondary Outcome: Adverse Effects & Adverse Drug Events

Study	Outcome measure	Effect measures	Result
Chrischilles, 2014 [20]	Medication adverse effects in past three months (patient self-report)	n (%)	Intervention: 100 (12.9)<br>Control: 33 (12)<br>*P* = .7883
Schnipper, 2012 [26]	Number of preventable or ameliorable ADEs per patient (Patient self-report survey, assessed by RA or blinded physician adjudicators)	Mean (SD)<br><br><br>Adjusted RR (95% CI)	Intervention: 0.16 (0.44)<br>Control: 0.14 (0.47)<br><br>1.21 (0.74 to 1.98)<br>*P* = .45
	Duration of ameliorable ADEs in days (Patient self-report survey, assessed by RA or blinded physician adjudicators)	Median (IQR)<br><br><br>Adjusted OR (95% CI)	Intervention: 5 (1-7)<br>Control: 6 (1-8)<br><br>0.64 (0.25 to1.66)<br>*P* = .35
Weingart, 2013 [28]	Number of ADEs (patient self-report telephone survey and review of electronic medical record)	Number of events (rate/100 patients)	Intervention: 98 (26.1)<br>Control: 93 (25.6)<br>*P* = .89
	Preventable ADEs (patient self-report telephone survey and review of electronic medical record)	Number of events (rate/100 patients)	Intervention: 6 (1.6)<br>Control: 2 (0.6)<br>*P* = .22
	Ameliorable ADEs (patient self-report telephone survey and review of electronic medical record)	Number of events (rate/100 patients)	Intervention: 24 (6.4)<br>Control: 19 (5.2)<br>*P* = .43
Mooney, 2017 [29]	Treatment impact	Average difference in severity points reduced for SCH vs UC	3.59 (P < 0.001)
	Adverse effects reduction	Number of symptoms reduced for SCH vs UC	10 of 11 symptoms (P: 0.025 to <0.001)

Supplemental Table 7: Results per outcome from included studies-- Secondary Outcome: Changes in Signs and Symptoms of Health Conditions

Cho, 2006 [19]	FBG (mmol/L) (bloodwork at follow-up)	Mean (SD)	Intervention: 8.51 (2.68)<br>Control: 7.87 (1.83)<br>t = 1.25, *P* = .22
	A1C (%) (bloodwork at follow-up)	Mean (SD)	Intervention: 6.7% (0.9%)<br>Control: 7.4 % (1.3%)<br>t = 2.8, *P* = .006
	Total cholesterol (mmol/L) (bloodwork at follow-up)	Mean (SD)	Intervention: 4.5 (0.67)<br>Control: 4.49 (0.76)<br>t = 0.062, *P* = .95
	Triglyceride (mmol/L) (bloodwork at follow-up)	Mean (SD)	Intervention: 1.16 (0.73)<br>Control: 1.28 (0.75)<br>t = 0.73 , *P* = .47
	HDL cholesterol (mmol/L) (bloodwork at follow-up)	Mean (SD)	Intervention: 1.37 (0.36)<br>Control: 1.26 (0.34)<br>t = 1.41, *P* = .16
Fiks, 2015 [21]	Days of school missed in the past month (patient/parent report of outcomes)	Mean (SD not reported)<br><br>n (%) children missing ≥1 day of school	Intervention: 0.6<br>Control: 1.9<br>*P* = .2<br><br>Intervention: 14 (52)<br>Control:16 (57)<br>*P* = .7
	Days of work missed in the past month (patient/parent report of outcomes)	Mean (SD not reported)<br><br>n (%) parents missing ≥1 day of work	Intervention: <0.1<br>Control: 1.5<br>*P* = .001<br><br>Intervention: 1 (4)<br>Control: 10 (37)<br>*P* = .07
	Nighttime symptoms (patient/parent report of outcomes)	Mean (SD)	Intervention: 85.0 (19.6)<br>Control 80.0 (21.3)<br>*P* = .9
	Daytime symptoms (patient/parent report of outcomes)	Mean (SD)	Intervention: 82.3 (17.5)<br>Control 68.7 (21.4)<br>*P* = .3
	Frequency of asthma flares (patient/parent report of outcomes)	Mean (SD)	Intervention: 1.4 (2.0)<br>Control: 3.8 (3.8)<br>*P* = .02
	Asthma symptoms while at best (patient/parent report of outcomes)	Mean (SD)	Intervention: 2.1 (2.1)<br>Control: 4.4 (5.3)<br>*P* = .1
	Number of poor/uncontrolled asthma episodes	Number of events (intervention only reported)	17 events in 13 children
	Functional limitations (patient/parent report of outcomes)	Mean (SD)	Intervention: 86.5 (20.0)<br>Control: 80.2 (24.1)<br>*P* = .4
Grant, 2008 [22]	HbA1C (patient self-report to PHR)	Mean % HcA1C	Intervention: 7.1<br>Control: 7.2<br>*P* = .45
	Patients at HbA1C goal (patient self-report to PHR)	n (%)	Intervention: 92 (73)<br>Control: 80 (68)<br>*P* = .53

Gustafson, 2012 [23]	Symptom free days (Asthma Control Questionnaire)	OR (95% CI)	0.18 (-0.88 to 1.60)<br>*P* = 1.00
	Asthma control (Asthma Control Questionnaire)	Mean change in intervention group minus mean change in control group (95% CI)	-0.31 (-0.56 to -0.06)<br>*P* = .01
Joseph, 2007 [24]	Symptom-nights (patient self-report follow-up survey)	Mean per two weeks (SD)<br><br>Adjusted RR (95% CI)	Intervention: 0.9 (2.3)<br>Control: 1.5 (2.5)<br>0.4 (0.2 to 0.8)<br>*P* = .009
	Symptom-days (patient self-report follow-up survey)	Mean per two weeks (SD)<br><br>Adjusted RR (95% CI)	Intervention: 2.1 (3.0)<br>Control: 2.8 (3.4)<br>0.5 (0.4 to 0.8)<br>*P* = .003
	Days of restricted activity (patient self-report follow-up survey)	Mean per two weeks (SD)<br><br>Adjusted RR (95% CI)	Intervention: 1.3 (2.2)<br>Control: 2.3 (3.4)<br>0.5 (0.3 to 0.8)<br>*P* = .02
	Days of changed plans (patient self-report follow-up survey)	Mean (SD)<br><br>Adjusted RR (95% CI)	Intervention: 0.4 (1.2)<br>Control: .6 (1.5)<br>0.5 (0.3 to 1.2)<br>*P* = .17
	Schooldays missed (patient self-report follow-up survey)	Mean per 30 days (SD)<br><br>Adjusted RR (95% CI)	Intervention: 0.4 (1.2)<br>Control: 1.2 (3.3)<br>0.3 (0.1 to 0.7)<br>*P* = .006
Joseph, 2013 [25]	Symptom-days (patient self-report follow-up survey)	 Mean (SD)<br><br>Adjusted RR (95% CI)	All patients:<br>Intervention: 3.9 (5.9)<br>Control: 5.2 (6.4)<br>0.8 (0.6 to 1.0)<br>*P* = .019<br><br>Moderate to severe asthma:<br>Intervention: 6.2 (7.7)<br>Control: 9.2 (8.1)<br>0.6 (0.5 to 0.9)<br>*P* = .013
	Symptom-nights (patient self-report follow-up survey)	 Mean (SD)<br><br>Adjusted RR (95% CI)	All patients:<br>Intervention: 2.7 (5.6)<br>Control: 2.8 (4.9)<br>(0.7 to 1.6)<br>*P* = .82<br><br>Moderate to severe asthma:<br>Intervention: 5.1 (6.6)<br>Control: 6.4 (7.9)<br>0.7 (0.4 to 1.2)<br>*P* = .210
	Schooldays missed (patient self-report follow-up survey)	 Mean (SD)<br><br>Adjusted RR (95% CI)	All patients:<br>Intervention: 2.6 (4.3)<br>Control: 3.1 (4.9)<br>0.8 (0.6 to 1.0)<br>*P* = .08<br><br>Moderate to severe asthma:<br>Intervention: 3.5 (5.6)

			Control: 5.1 (7.0)<br>0.5 (0.3 to 0.8)<br>*P* = .009
	Schooldays missed because of asthma (patient self-report follow-up survey)	Mean (SD)<br><br>Adjusted RR (95% CI)	<u>All patients:</u><br>Intervention: 0.8 (2.1)<br>Control: 1.4 (3.9)<br>0.8 (0.5 to 1.2)<br>*P* = .25<br><br><u>Moderate to severe asthma:</u><br>Intervention: 1.3 (2.6)<br>Control: 3.3 (6.6)<br>0.4 (0.2 to 0.8)<br>*P* = .007
	Days of restricted activity (patient self-report follow-up survey)	Mean (SD)<br><br>Adjusted RR (95% CI)	<u>All patients:</u><br>Intervention: 3.2 (5.5)<br>Control: 4.2 (6.0)<br>0.8 (0.6 to 1.1)<br>*P* = .14<br><br><u>Moderate to severe asthma</u>:<br>Intervention: 5.3 (7.4)<br>Control: 7.1 (7.6)<br>0.6 (0.4 to 0.9)<br>*P* = .025
	Uncontrolled asthma two or more days per week over 30 days (patient self-report follow-up survey)	n (%)<br><br>Adjusted OR (95% CI)	Intervention: 23 (11)<br>Control: 46 (21)<br>0.5 (0.2 to 0.8)<br>*P* = .006
	Three or more symptom-nights (patient self-report follow-up survey)	n (%)<br><br>Adjusted OR (95% CI)	Intervention: 50 (25)<br>Control: 68 (31)<br>0.6 (0.4 to 1.0)<br>*P* = .074
	More than two schooldays missed (patient self-report follow-up survey)	n (%)<br><br>Adjusted OR (95% CI)	Intervention: 68 (33)<br>Control: 89 (41)<br>0.7 (0.4 to 1.1)<br>*P* = .09
	More than two schooldays missed due to asthma (patient self-report follow-up survey)	n (%)<br><br>Adjusted OR (95% CI)	Intervention: 26 (13)<br>Control: 32 (15)<br>0.8 (0.5 to 1.5)<br>*P* = .52
	More than four days of restricted activity (patient self-report follow-up survey)	n (%)<br><br>Adjusted OR (95% CI)	Intervention: 39 (19)<br>Control: 68 (31)<br>Adjusted OR (95% CI): 5 (0.3 to 0.9)<br>*P* = .01
	More than four days of changed plans (patient self-report follow-up survey)	n (%)<br><br>Adjusted OR (95% CI)	Intervention: 20 (10)<br>Control: 24 (11)<br>0.8 (0.4 to 1.7)<br>*P* = .58
Simon, 2011 [27]	Depression score (Symptom Checklist)	Mean (SD)	Intervention: 0.95 (0.71)<br>Control: 1.17 (0.81)<br>t = 2.05, *P* = .043,
Ahmed, 2016 [30]	Asthma Control status at 6 months	n (%)	Intervention (observed): 45(17)

		OR (95% CI)	Control (observed): 45(16)<br>0.62 (0.16 to 2.33)<br><br>Intervention (imputed): 46(18)<br>Control (imputed): 51(19)<br>0.94 (0.33 to 2.71)
Karhula, 2015 [31]	HbA1C goal (measured at follow-up)	Change (95% CI)	Intervention: 0.04 (-0.09, 0.17)<br>Control: 0.18 (-0.02, 0.35)
	Waist circumference, between-group difference	Change (95% CI)	-1.711 (-3.042 to -0.38)<br>*P = 0.01*
Carlsen, 2017 [32]	Trough IFX concentration – controlled for treatment intervals	Change (95% CI)	-2.19 (-5.37 to 0.99)<br>*P = 0.18*

Supplemental Table 8: Results per outcome from included studies-- Secondary Outcome: Health Services Utilization

Study	Outcome measure	Effect measures	Result
Fiks, 2015 [21]	Hospitalizations (patient/parent self-report)	Number of events<br><br>OR (95% CI)	Intervention: 0<br>Control: 2<br>0.33 (0.013 to 8.56)<br>*P* = .51
	ED visits (patient/parent self-report)	Number of events<br><br>OR	Intervention: 3<br>Control: 9<br>0.29 (0.053 to 1.60)<br>*P* = .16
	Asthma specialist visits (patient/parent self-report)	Number of events<br><br>OR	Intervention: 11<br>Control: 21<br>0.56 (0.18 to1.71)<br>*P* = .31
	Asthma primary care visits (patient/parent self-report)	Number of events<br><br>OR	Intervention: 29<br>Control: 41<br>0.80 (0.26 to 2.46)<br>*P* = .70
Joseph, 2007 [24]	Asthma-related ED visits (patient self-report)	Mean per 12 months (SD)<br><br>Adjusted RR (95% CI)	Intervention: 0.5 (2.0)<br>Control: 0.8 (1.9)<br>0.5 (0.3-1.3)<br>*P* = .08
	Asthma-related hospitalizations (patient self-report)	Mean per 12 months (SD)<br><br>Adjusted RR (95% CI)	Intervention: 0.2 (0.6)<br>Control: 0.6 (2.0)<br>RR: 0.2 (0.2-0.9)<br>*P* = .01
Joseph, 2013 [25]	Asthma-related ED visits (patient self-report)	Mean (SD)<br><br>Adjusted RR (95% CI)	<u>All patients:</u><br>Intervention: 0.9 (2.1)<br>Control: 0.9 (2.4)<br>1.0 (0.7 to 1.4)<br>*P* = .92<br><br><u>Moderate to severe asthma:</u><br>Intervention: 1.5 (3.4)<br>Control: 1.7 (3.7)<br>1.0 (0.5 to 2.0)<br>*P* = .95
	Asthma-related hospitalizations (patient self-report)	Mean (SD)<br><br>Adjusted RR (95% CI)	<u>All patients:</u><br>Intervention: 0.3 (1.2)<br>Control: 0.3 (1.0)<br>1.2 (0.5 to 2.6)<br>*P* = .66<br><br><u>Moderate to severe asthma:</u><br>Intervention: 0.3 (0.8)<br>Control: 0.5 (1.1)<br>0.6 (0.2 to 2.2)<br>*P* = .47
Simon, 2011 [27]	Participants making any specialty mental health visits (EMR data)	n (%)<br><br>Chi square, df	Intervention: 34 (32)<br>Control: 32 (31)<br>0.01, 1.<br>*P* = .91
	Mental health visits for	Mean (SD)	Intervention: 0.4 (1.4)

	medication management (EMR data)		Control: 0.3 (1.5)<br>*P* = .18
Weingart, 2013 [28]	Primary care appointments (patient self-report and EMR data)	Number of events (rate/100 patients)	Intervention: 579 (154.4)<br>Control: 556 (153.2)<br>*P* = .90
	Specialist appointments (patient self-report and EMR data)	Number of events (rate/100 patients)	Intervention: 824 (219.7)<br>Control: 675 (186.0)<br>*P* = .19
	ED visits (patient self-report and EMR data)	Number of events (rate/100 patients)	Intervention: 31 (8.3)<br>Control: 34 (9.4)<br>*P* = .72
	Hospital admissions (patient self-report and EMR data)	Number of events (rate/100 patients)	Intervention: 22 (5.9)<br>Control: 25 (6.9)<br>*P* = .62
	Inpatient days (patient self-report and EMR data)	Number of events (rate/100 patients)	Intervention: 54 (14.4)<br>Control: 86 (23.7)<br>*P* = .23

Supplemental Table 9: Results per outcome from included studies—Secondary Outcome: Patient overall health status

Study	Outcome measure	Effect measures	Result
Joseph, 2007 [24]	QoL cumulative score	Mean (SD)<br><br>Adjusted RR (95% CI)	Intervention: 5.3 (1.3)<br><br>Control: 5.0 (1.5)<br><br>1.2 (0.9 to 1.6)<br><br>*P* = .35
Ahmed, 2016 [30]	Mini-Asthma Quality of Life Questionnaire (MAQLQ)	Mean (SD)<br><br>Mean Change (95% CI)	Intervention (observed): 5.6(1.2)<br>Control (observed): 5.2(1.2)<br>0.31 (-0.21 to 0.84)<br><br>Intervention (imputed): 5.3(1.3)<br>Control (imputed): 5.1(1.2)<br>0.22 (-0.34 to 0.78)
Karhula, 2015 [31]	QoL General Health score	Heart disease patients<br>Change (95% CI)	Intervention: 2.60 (0.36, 4.84)<br>Control: 0.56 (-2.93, 4.05)<br>Difference = 1.77 (-2.06, 5.61)<br>*P=0.36*
	QoL General Health score	Diabetes patients<br>Change (95% CI)	Intervention: 3.47 (1.04, 5.89)<br>Control: 1.34 (-1.48, 4.17)<br>Difference: 2.34 (-1.72, 6.41)<br>*P=0.26*
Carlsen, 2017 [32]	Health-Related Quality of Life	Mean (SD)	First score: 150.0 (15.6)<br>Last score: 148.8 (18.0)<br>Difference: 2.2 (16.6)<br>*P = 0.63*

Supplemental Table 10: Results per outcome from included studies—Secondary Outcome: Patient satisfaction with health care

Study	Outcome measure	Effect measures	Result
Fiks, 2015 [21]	Satisfaction with asthma care (patient questionnaire)	N/A	“There were no significant differences between study arms in satisfaction with asthma care (data not shown)”[15]
Simon, 2011 [27]	Quality of antidepressant treatment (patient questionnaire)	n (%) patients very satisfied	Intervention: 56 (53)<br>Control: 31 (33)<br>*P* = .004

Supplemental Table 11: Results of Subgroup Analyses: Age

Subgroup	Studies included in subgroup	Results
Children and teenagers	21, 23-25, 32	Evidence for the effect of ehealth tools on asthma-related hospitalizations was mixed, with two studies reporting no effect of intervention [21, 25], and one study reporting a significant decrease in the intervention group [24]. Evidence for the outcome of improvement of symptoms of health conditions was generally positive. Fiks et al. [21] found significantly less frequent asthma flare-ups in the intervention group, and Gustafson et al. [23] found a significant improvement in asthma control in the intervention. Joseph et al. [24, 25] found less asthma symptom-nights in the intervention group. Joseph et al. [24] found a significant increase of children with rescue inhalers in the intervention group. Carlsen et al. [32] found a significant decrease in number of infliximab infusions required in patients using the ehealth tool. Overall, there is a positive trend showing that ehealth interventions may improve asthma symptoms and control, and there is a small amount of evidence that they may help decrease asthma-related hospitalizations.
Elderly (65+)	20	No significant differences were seen between active and control groups for any outcomes with the exception of inappropriate use ≥2 NSAID medications, which was significantly lower in the intervention group.
Adults	19, 22, 27, 29-31	Comparative evidence from RCTs is mixed for the primary outcome of changes made to medication regimens, with one study finding no differences between groups [19], and two studies finding higher numbers of medication changes in the intervention groups [22, 27]. Simon et al. [27] reported an improvement in patient satisfaction with depression care favouring use of ehealth tools. Mooney et al. [29] found a significant decrease in 10 of 11 measured adverse effects as a result of chemotherapy in the intervention group relative to usual care. Ahmed et al. [30] found a significant improvement in depression symptoms in asthmatic patients. Karhula et al. [31] found no significant changes in signs or symptoms of chronic disease or in overall health status.

Supplemental Table 12: Results of Subgroup Analyses: Conditions

Subgroup	Studies included in subgroup	Results
Asthma	21, 23-25, 30	There is evidence that ehealth tools may have the potential to reduce symptoms of asthma, frequency of asthma flare-ups, and number of days of school or work missed due to asthma [21, 23-25]. They may also promote better asthma control, availability and use of rescue inhalers, and may have the potential to improve asthma symptoms in vulnerable groups (i.e. African-American adolescents living in urban centres [24, 25]. One study that focused on asthma found no statistically significant difference in asthma-related outcome, however it found a significant improvement in symptoms of depression at six months in asthmatic patients [30].
Diabetes	19, 22, 31	Use of ehealth tools for patient reporting of symptoms and adverse effects may have the potential to influence medication changes in diabetics [31]. The use of ehealth tools to reduce HbA1C was mixed, making it difficult to draw conclusions [19, 22, 31].

Supplemental Table 13: Results of Subgroup Analyses: Intervention Type

**Subgroup**	**Studies included in subgroup**	**Results**
ehealth tool only	20-22, 27-28, 32	Of the three studies in this group investigating changes in medications [20, 22, 27], all found at least one significant increase in medication changes favouring ehealth tools. Of the three studies in this group investigating improvements in signs and symptoms [21, 22, 27], only Grant et al. [22] did not find at least one significant improvement in outcomes.
ehealth tool plus clinician support/case management	19, 23-26, 29-31	Two of three studies in this group that measured self-management or self-efficacy [23, 26, 30] found positive significant effects for at least one outcome. One study measured medication reconciliation and recommendation, and it found two positive significant outcomes [31]. All six studies measuring improvements in signs and symptoms found at least one positive significant improvement [19, 23-26, 29-31].

Supplemental Table 14: Results of Subgroup Analyses: Linkage to EMRs

**Subgroup**	**Studies included in subgroup**	**Results**
Linked	21, 22, 26-31	Of the two studies that measured medication reconciliation and recommendation, one found two positive significant outcomes [26]. Both studies with this functionality measured ≥1 positive significant increase in medication changes [22, 27]. Five studies measured changes in signs and symptoms; four found ≥1 positive significant outcomes [21, 27, 30, 31], and one found no significant outcomes [28]. Two studies that measured patient overall health status both found no significant difference in this outcome [30, 31].
Unlinked	19, 20, 23-25, 32	Four studies measured changes in medication; three found ≥1 positive significant outcomes [20, 24, 32], and one found no significant outcomes [19]. Five studies measured changes in signs and symptoms of which four found >1 positive significant outcomes [20, 23-25], and one found no significant difference in outcomes [32].

Supplemental Table 15: Results of Subgroup Analyses: Direct Clinician Access to Patient Self-Reports

Subgroup	Studies included in subgroup	Results
Direct access to self-reports	19, 21-23, 26, 28-32	Four studies measured patient self-management and self-efficacy; two found ≥1 positive significant outcomes [23, 26], and two found no significant outcomes [21, 30]. One study measured medication reconciliation and recommendation, and it found two positive significant outcomes [26]. Seven studies measured changes in signs and symptoms; five found >1 positive significant outcomes [19, 21, 23, 30, 31], and two found no significant outcomes [22, 32].
No access to self-reports	20, 23, 25, 27	Three studies measured changes in medication, and all three had ≥1 positive significant outcome [20, 24, 27]. Three studies measured changes in signs and symptoms, and all found >1 positive significant outcomes [24, 25, 27]. One study measured patient satisfaction, and it found one positive, significant outcome [27].

Supplemental Table 16: Results of Subgroup Analyses: Direct Patient-Provider Communication

Subgroup	Studies included in subgroup	Results
With communication functionality	19, 22, 23, 27-29, 30-31	Three studies measured changes in medication; two found ≥1 positive significant outcomes [22, 27], and one found no significant outcomes [19]. Six studies measured changes in signs and symptoms; five found >1 positive significant outcomes [19, 23, 27, 30, 31], and one found no significant outcomes [22]. Of three studies with this functionality that reported on self-management and self-efficacy [23, 26], two found a positive significant improvement [23, 26] and one found no significant improvement [30]. One study measured medication reconciliation and recommendation, and it found two positive significant outcomes [26]. Two studies that reported no effect of intervention for self-management and self-efficacy did not utilize this functionality [20, 21]. One study measured patient satisfaction, and it found one positive, significant outcome [27].
Without communication functionality	20, 21, 24, 25, 29	Two of these studies measured changes in medication, and both found ≥1 positive significant outcome [20, 24]. Three studies measured changes in signs and symptoms, and all found >1 positive significant outcomes [21, 24, 25].
