# Supplementary material for: The Use and Effects of Electronic Health Tools for Patient Self-Monitoring and Reporting of Outcomes Following Medication Use: Systematic Review
Source: J Med Internet Res. 2018 Dec 18;20(12):e294. doi: 10.2196/jmir.9284 (PMC6315271; doi:10.2196/jmir.9284)
Supplement: Multimedia Appendix 5 [file jmir_v20i12e294_app5.pdf]

Multimedia Appendix V. Breakdown of study results by outcome

Outcome	Cho, 2006 [19]	Chrischilles, 2014 [20]	Fiks, 2015 [21]	Grant, 2008 [22]	Gustafson, 2012 [23]	Joseph, 2007 [24]	Joseph, 2013 [25]	Schnipper, 2012 [26]	Simon, 2011 [27]	Weingart, 2013 [28]	Mooney, 2017 [29]	Ahmed, 2016 [30]	Karhula, 2015 [31]	Carlsen, 2017 [32]
Changes in use of medications	**N**	**P**	**D**	**P**		**P**			**P**					**P**
Changes in signs and symptoms of chronic disease	**P**		**P**	**N**	**P**	**P**	**P**		**P**			**P**	**P**	**N**
Patient self-management and self-efficacy		**N**	**N**		**P**			**P**				**P**		
Medication use behaviour		**N**			**N**	**N**								
Medication reconciliation and recommendations for change	**D**	**N**						**P**						
Identification of adverse effects & ADEs		**N**	**D**					**N**		**N**	**P**			**D**
Health service utilization			**N**			**P**	**N**		**N**	**N**		**N**		
Patient overall health status						**N**						**N**	**N**	**N**
Patient satisfaction with health care			**N**						**P**					**D**

P is used to demonstrate at least one significant effect on outcomes.

N is used to demonstrate no significant effects on outcomes.

D is used to demonstrate descriptive data only.
